# Type 2 Diabetes Alters Intracellular Ca^2+^ Handling in Native Endothelium of Excised Rat Aorta

**DOI:** 10.3390/ijms21010250

**Published:** 2019-12-30

**Authors:** Roberto Berra-Romani, Alejandro Guzmán-Silva, Ajelet Vargaz-Guadarrama, Juan Carlos Flores-Alonso, José Alonso-Romero, Samuel Treviño, Josué Sánchez-Gómez, Nayeli Coyotl-Santiago, Mario García-Carrasco, Francesco Moccia

**Affiliations:** 1 Laboratory of Cardiovascular Physiology, Biomedicine School, Faculty of Medicine, Benemérita Universidad Autónoma de Puebla, Puebla 72410, Mexico; aleckz.guzman@gmail.com (A.G.-S.); oublitte_15@hotmail.com (J.A.-R.); nayevader@gmail.com (N.C.-S.); 2 Faculty of Medicine, Benemérita Universidad Autónoma de Puebla, Puebla 72410, Mexico; ajeletvargaz7@hotmail.com (A.V.-G.); k.o.k._1967@hotmail.com (J.S.-G.); mgc30591@yahoo.com (M.G.-C.); 3 Centro de Investigación Biomédica de Oriente, Instituto Mexicano del Seguro Social, Puebla 74360, Mexico; flores_alonso_jc@hotmail.com; 4 Facultad de Ciencias Químicas, Benemérita Universidad Autónoma de Puebla, Puebla 72540, Mexico; samuel_trevino@hotmail.com; 5 Laboratory of General Physiology, Department of Biology and Biotechnology “Lazzaro Spallanzani”, University of Pavia, 27100 Pavia, Italy

**Keywords:** Type 2 diabetes mellitus, intact endothelium, intracellular calcium, Fura-2, sarco-endoplasmic reticulum Ca^2+^-ATPase, Na^+^-Ca2^+^ exchanger, plasma membrane Ca^2+^-ATPase

## Abstract

An increase in intracellular Ca^2+^ concentration ([Ca^2+^]_i_) plays a key role in controlling endothelial functions; however, it is still unclear whether endothelial Ca^2+^ handling is altered by type 2 diabetes mellitus, which results in severe endothelial dysfunction. Herein, we analyzed for the first time the Ca^2+^ response to the physiological autacoid ATP in native aortic endothelium of obese Zucker diabetic fatty (OZDF) rats and their lean controls, which are termed LZDF rats. By loading the endothelial monolayer with the Ca^2+^-sensitive fluorophore, Fura-2/AM, we found that the endothelial Ca^2+^ response to 20 µM and 300 µM ATP exhibited a higher plateau, a larger area under the curve and prolonged duration in OZDF rats. The “Ca^2+^ add-back” protocol revealed no difference in the inositol-1,4,5-trisphosphate-releasable endoplasmic reticulum (ER) Ca^2+^ pool, while store-operated Ca^2+^ entry was surprisingly down-regulated in OZDF aortae. Pharmacological manipulation disclosed that sarco-endoplasmic reticulum Ca^2+^-ATPase (SERCA) activity was down-regulated by reactive oxygen species in native aortic endothelium of OZDF rats, thereby exaggerating the Ca^2+^ response to high agonist concentrations. These findings shed new light on the mechanisms by which type 2 diabetes mellitus may cause endothelial dysfunction by remodeling the intracellular Ca^2+^ toolkit.

## 1. Introduction

Endothelial cells form a monolayer comprising the innermost lining of blood vessels and maintain cardiovascular (CV) homeostasis by regulating vascular tone, permeability, coagulation, immune response and by controlling vascular growth and repair [1,2]. Therefore, endothelial dysfunction is regarded as a crucial driver of multiple life-threatening CV disorders, such as hypertension, coronary artery disease, myocardial infarction, and stroke [3]. Type 2 diabetes mellitus is regarded as a health problem of epidemic proportions, with a prevalence of 2–5% in western countries, and is predicted to rise due to the global changes in lifestyle and population aging [4,5]. CV disorders represent the major responsible for the shorter longevity of diabetic patients, who are at about a 60% increased risk of early mortality, and the adverse effects of diabetes are amplified by hypertension and obesity [6]. Endothelial dysfunction may precede the development and contribute to the vascular complications of type 2 diabetes mellitus [4,5,7,8], including coronary artery disease, peripheral arterial disease, stroke (macrovascular) and diabetic retinopathy, nephropathy and neuropathy (microvascular). For instance, it has been shown that endothelium-dependent vasodilation is impaired in type 2 diabetes mellitus due to the reduction in nitric oxide bioavailability and the increase in endothelin-1 production [4,5], thereby resulting in hypertension and atherosclerosis. Moreover, sprouting angiogenesis, which drives the formation of new capillaries from pre-existing microvessels, is exacerbated in the retina, thereby resulting in retinopathy [9].

An increase in intracellular Ca^2+^ concentration ([Ca^2+^]_i_) is crucial to fine tune endothelial cell functions [10,11,12]. For instance, endothelial Ca^2+^ signals drive the release of many vasodilators, including nitric oxide, prostacyclin, arachidonic acid metabolites and hydrogen sulfide, and recruit Ca^2+^-activated K^+^ channels to stimulate endothelium-dependent hyperpolarization [13,14,15,16]. In addition, an increase in endothelial [Ca^2+^]_i_ leads to the production of the vasoconstrictor prostanoids, thromboxane A2, prostaglandin H2 and endothelin-1 [14,17,18]. Vasoactive agonists bind to specific G-protein coupled receptors to engage phospholipase Cβ, a membrane-bound enzyme that cleaves inositol-1,4,5-trisphosphate (InsP_3_) from its precursor phosphatidylinositol-4,5-bisphosphate [14,15]. The following Ca^2+^ response typically consists of an initial Ca^2+^ peak, which is mediated by InsP_3_-induced Ca^2+^ release from the endoplasmic reticulum (ER), followed by a prolonged influx of Ca^2+^, known as a plateau [19,20]. This plateau phase is mediated by store-operated Ca^2+^ entry (SOCE), that represents one of the major Ca^2+^-entry pathways in endothelial cells and is activated upon depletion of the InsP_3_-sensitive ER Ca^2+^ store [11,19,20]. The clearance of [Ca^2+^]_i_ after agonist stimulation is achieved by several Ca^2+^-transporting systems, such as plasma membrane Ca^2+^-ATPase (PMCA), Na^+^/Ca^2+^ exchanger (NCX) and sarco-endoplasmic reticulum Ca^2+^-ATPase (SERCA) [21,22,23,24]. In addition, InsP_3_-dependent ER Ca^2+^ release may be buffered by closely apposed mitochondria, which could regulate local Ca^2+^ concentration at the mitochondria-associated ER membranes (MAMs), thereby favoring [25] or inhibiting [26] InsP_3_-dependent Ca^2+^ events. Dysregulation of the endothelial Ca^2+^ toolkit was observed in severe pathological conditions, including cancer [27,28], primary myelofibrosis [29], and Alzheimer’s disease [30]. Furthermore, endothelial Ca^2+^ handling may be compromised by the chronic exposure to CV risk factors, such as atherosclerosis [31], aging [32], oxidative stress [33], and inflammation [34]. Nevertheless, no study has thoroughly investigated the impairment of endothelial Ca^2+^ signaling in an animal model of type 2 diabetes mellitus.

Obese Zucker Diabetic Fatty (OZDF) rats have been widely employed as a type 2 diabetes mellitus model, which arises when the animals are fed with a high-energy diet as a consequence of a homozygous mutation (fa/fa) in the leptin hormone receptor [35]. OZDF rats develop obesity, hyperlipidemia, hypertension, hyperglycemia and insulin resistance, and therefore provide a suitable model to investigate how diabetes affects endothelial Ca^2+^ signaling in situ. The intact endothelium of excised rat aortae provides an ideal preparation for investigating endothelial Ca^2+^ handling, as the intracellular Ca^2+^ toolkit of native endothelium may differ from that described in cultured endothelial cells [36,37,38]. In the present study, we evaluated for the first time whether type 2 diabetes affects endothelial Ca^2+^ signaling in situ by exploiting rat aortas excised from OZDF rats and age-matched lean Zucker Diabetic Fatty rats (LZDF). We demonstrated that agonist-induced increase in [Ca^2+^]_i_ is dramatically enhanced in association with the down-regulation of SERCA activity in native endothelium of excised rat aorta of OZDF rats. These findings shed novel light on the mechanisms whereby diabetes causes endothelial injury and hint at the endothelial Ca^2+^ toolkit as a novel target to prevent CV complications in diabetic patients.

## 2. Results

### 2.1. Somatic and Biochemical Characteristics of OZDF Rats

The somatic and biochemical parameters obtained from 2–3-month-old littermate LZDF and OZDF rats are shown in Table 1. OZDF rats presented a 70% increase in body mass (weight) compared with their control, i.e., LZDF rats. This increase in weight was not due to a larger size of OZDF rats, since the measurements of the distance between the base of the tail and the tip of the nose (length) did not show any significant difference between the two groups of animals (*p* < 0.05). Indeed, the abdominal circumference and the body mass index (BMI) increased by 35% and 57%, respectively, in OZDF rats. In addition, we found that the epididymal fat weight from OZDF rats was 4 times higher than that obtained in LZDF rats. Taken together, these data prove the obese phenotype of the OZDF rat group.

The biochemical results, reported in Table 1, confirm other characteristics of the OZDF rat model: hyperlipidemia. Obese rats (OZDF) presented an increase of ≈46% in total cholesterol, ≈200% in the very low-density lipoprotein (VLDL) and ≈340% in triglyceride levels compared to LZDF rats. These results denote a clear alteration in the regulation of lipids in the obese-diabetic rat OZDF. Non-significant statistical differences were found on high-density lipoprotein cholesterol (HDL) and low-density lipoprotein cholesterol (LDL) blood levels in both experimental groups (*p* < 0.05).

**Figure 1:**
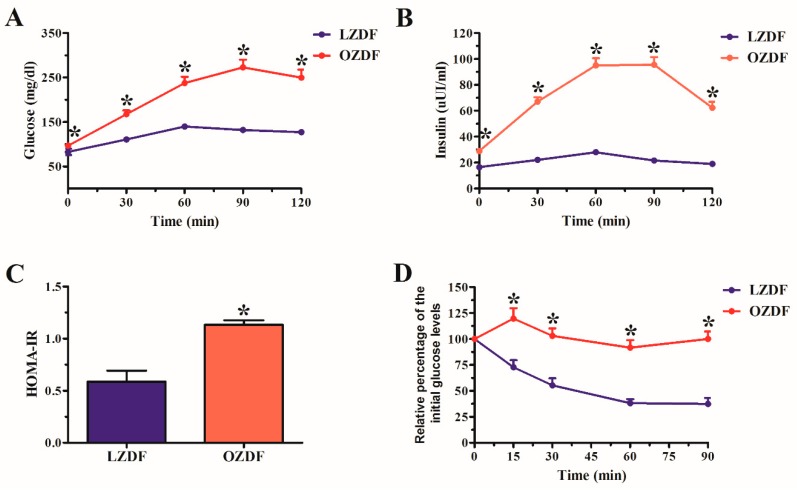
(**A**) Oral glucose tolerance test (OGTT). (**B**) Insulin concentration. (**C**) Homeostasis model assessment insulin resistance (HOMA-IR). (**D**) Insulin tolerance test (ITT). The values represent the mean ± SE (standard error). Data were compared using the Student’s *t*-test with a minimum significance value of 0.05 (*p* value). The asterisk indicates the significant differences observed when comparing the OZDF vs LZDF group. Analysis was performed with a n of 7 rats for the LZDF group and 7 rats for the OZDF group.

Figure 1 shows the results of oral glucose tolerance test (OGTT) (see Material and Methods), in which the fasting glucose was 82.7 ± 7.05 mg/dL in LZDF rats and 96.57 ± 1.688 mg/dL in OZDF rats (*p* < 0.05). After glucose loading, significant differences also were observed in the glucose tolerance in the OZDF group at 30, 60, 90 and 120 min corresponding to increases of 52%, 70%, 107%, and 97%, respectively (Figure 1A). Similarly, insulin concentration shows significant differences in OZDF rats, in both fasting and later of the glucose load hyperinsulinemia was observed, that corresponding to 75%, 203%, 239%, 341, and 228% at 0, 30, 60, 90 and 120 min (Figure 1B). It is known that high insulin levels lead to development to insulin resistance; thus, the homeostasis model assessment to evaluate insulin resistance (HOMA-IR) was carried out. The results show an increase of 93% in OZDF rats in relation to LZDF group (Figure 1C). The insulin resistance is linked to a low hormone tolerance. Therefore, we performed an insulin tolerance test (ITT), in which we observed that the percentage of the blood glucose presents significant changes between groups (Figure 1D). The LZDF rats showed a percentage decrease in the glucose that corresponded to 64%, 86%, 140%, and 167% at 15, 30, 60 and 90 min, respectively. Meanwhile, in the OZDF rats the glucose percentage increased by 20% at 15 min after insulin administration, while consecutive analysis times showed close values at 100%. This finding indicates that no changes were observed in plasmatic glucose, which suggests a low response to insulin or severe insulin resistance.

### 2.2. The Ca^2+^ Response to Adenosine Triphosphate (ATP) is Enhanced in Native Aortic Endothelium of Diabetic Rats

**Figure 2:**
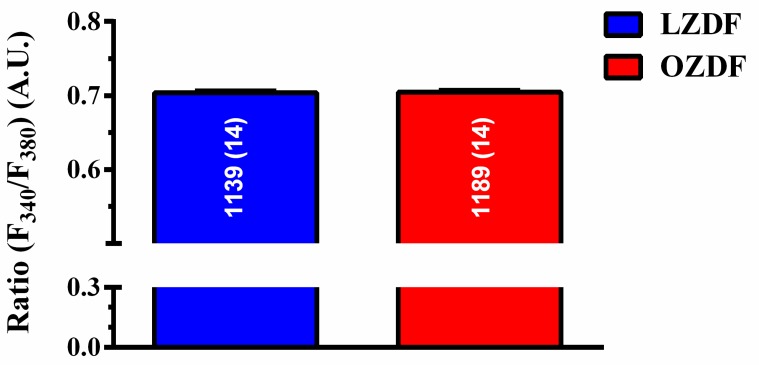
Resting [Ca^2+^]_i_ is not altered in native aortic endothelium of obese Zucker diabetic fatty rats. Ratiometric measurement (Ratio F340/F380) of resting Fura-2 fluorescence revealed that basal Ca^2+^ levels, expressed as mean ± SE, were not significantly different in OZDF as compared to LZDF rats. A.U. (arbitrary units).

Preliminary recordings carried out by loading the native endothelium in situ in rat aorta with the Ca^2+^-sensitive fluorophore, Fura-2/AM, revealed that basal Fura-2 fluorescence (Ratio F340/F380) was not statistically different in OZDF (0.7023 ± 0.0011, *n* = 1189 cells from 14 rats) vs LZDF rats (0.7018 ± 0.0013, *n* = 1139 cells from 14 rats) *p* > 0.05 (Figure 2).

The nucleotide adenosine triphosphate (ATP) has been widely employed to induce intracellular Ca^2+^ signals in the intact endothelium of excised rat aorta [38,39,40]. We have previously shown that the Ca^2+^ response to ATP was triggered by purinergic P_2Y1_ and P_2Y12/13_ receptors [36], which are coupled to phospholipase Cβ and InsP_3_-dependent Ca^2+^ release. The initial Ca^2+^ peak was followed by a sustained plateau phase that was larger at high (300 µM) than low (20 µM) ATP concentration, and was mainly due to SOCE activation [36,39]. Therefore, in order to assess whether type 2 diabetes mellitus impacts endothelial Ca^2+^ machinery, rat aortic rings from the same vessel were challenged with both low, i.e., 20 µM, and high, i.e., 300 µM, doses of ATP. The rationale behind this approach was that remodeling of the intracellular Ca^2+^ machinery could be more evident when a larger amount of Ca^2+^ was introduced into the cytosol by a higher agonist concentration. For instance, a subtle change in ER Ca^2+^ concentration could be unmasked by massive stimulation of InsP_3_Rs at 300 µM ATP. Moreover, any alteration in the Ca^2+^ clearing system (e.g., slowing down of Ca^2+^ removal from the cytosol) could lead to a dramatic accumulation of cytosolic free Ca^2+^ at high ATP concentration, when it is more difficult for pumps and transporters to cope with the enhanced Ca^2+^ load [41,42].

**Figure 3:**
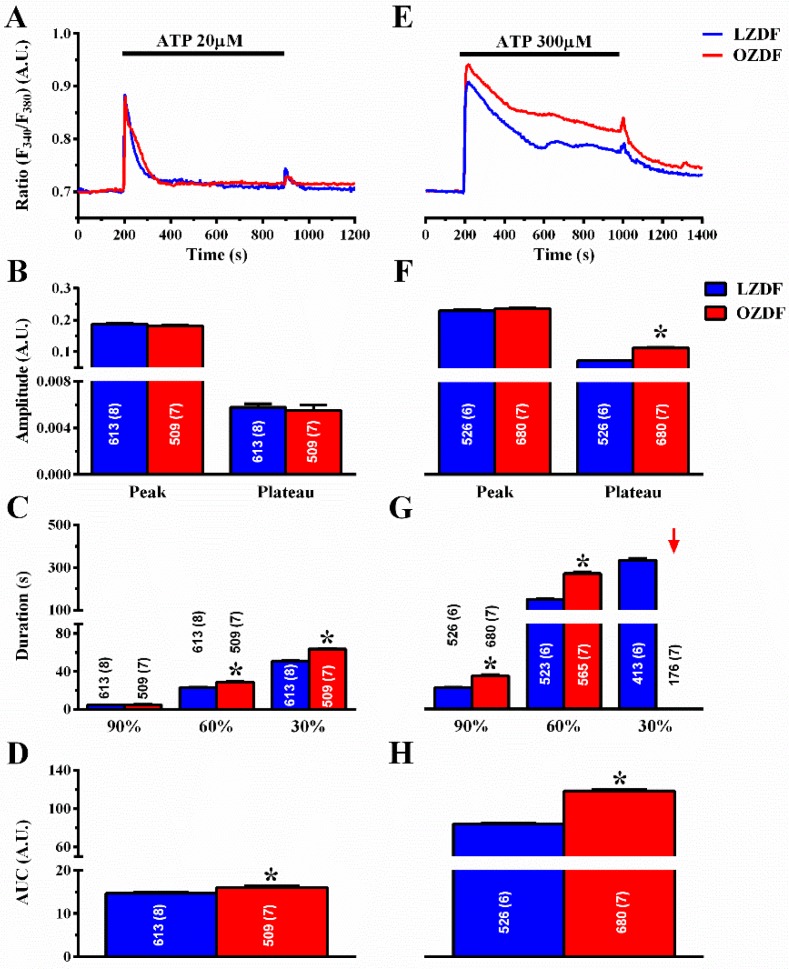
The Ca^2+^ response to low and high doses of the physiological agonist, ATP, is increased in native aortic endothelium of obese Zucker diabetic fatty rats. (**A**) Representative recordings of the Ca^2+^ response to 20 µM ATP in LZDF (blue line) and OZDF rats (red line). (**B**) Mean ± SE of the peak and plateau phase of the Ca^2+^ response to 20 µM ATP in LZDF and OZDF rats. (**C**) Mean ± SE of the duration of the Ca^2+^ response to 20 µM ATP in LZDF and OZDF rats. The asterisk indicates *p* < 0.05. (**D**) Mean ± SE of the AUC of the Ca^2+^ response to 20 µM ATP in LZDF and OZDF rats. (**E**) Representative recordings of the Ca^2+^ response to 300 µM ATP in LZDF and OZDF rats. (**F**) Mean ± SE of the peak and plateau phase of the Ca^2+^ response to 300 µM ATP in LZDF and OZDF rats. The asterisk indicates *p* < 0.05. (**G**) Mean ± SE of the duration of the Ca^2+^ response to 300 µM ATP in LZDF and OZDF rats. The asterisk indicates *p* < 0.05. Red arrow indicates that the Ca^2+^ signal failed to reach the clearing rate to the 30% of the initial peak amplitude in OZDF rats. (**H**) Mean ± SE of the AUC of the Ca^2+^ response to 300 µM ATP in LZDF and OZDF rats. The asterisk indicates *p* < 0.05, Mann-Whitney test.

Figure 3A shows that, when the excised endothelium was stimulated with 20 µM ATP in the presence of extracellular Ca^2+^, there was no difference in the amplitude of the initial Ca^2+^ peak and of the following plateau phase (Figure 3B). However, the duration of the Ca^2+^ response at 60% and 30% of the initial Ca^2+^ response (Figure 3C) and the area under the curve (AUC) (Figure 3D) were higher in OZDF rats as compared to LZDF rats. When the excised endothelium was stimulated with 300 µM ATP (Figure 3E), the amplitude of the plateau phase (Figure 3F, right) and the AUC (Figure 3H) were significantly (*p* < 0.05) increased in OZDF as compared to LZDF rats. Moreover, a significant increase was found in the times to 90 and 60% decay of the initial Ca^2+^ response in OZDF rats (*p* < 0.05) (Figure 3G). Notably, the Ca^2+^ signal clearly decayed to 30% of the initial peak amplitude in ≈78% (413 out of 526 cells) of LZDF rats, while it failed to do so in ≈26% (176 of 680 cells) of OZDF rats. For this reason, the late clearing rate of OZDF rats was not displayed (red arrow) and compared with LZDF values in Figure 3G. These data, therefore, indicate that type 2 diabetes mellitus alters the endothelial Ca^2+^ response evoked by both low, i.e., 20 µM and strong, i.e., 300 µM, stimulation by a physiological agonist, such as ATP.

### 2.3. The Rate of Decay of Intracellular Ca^2+^ Release Is Shortened and SOCE Amplitude Is Reduced in Native Aortic Endothelium of OZDF Rats

**Figure 4:**
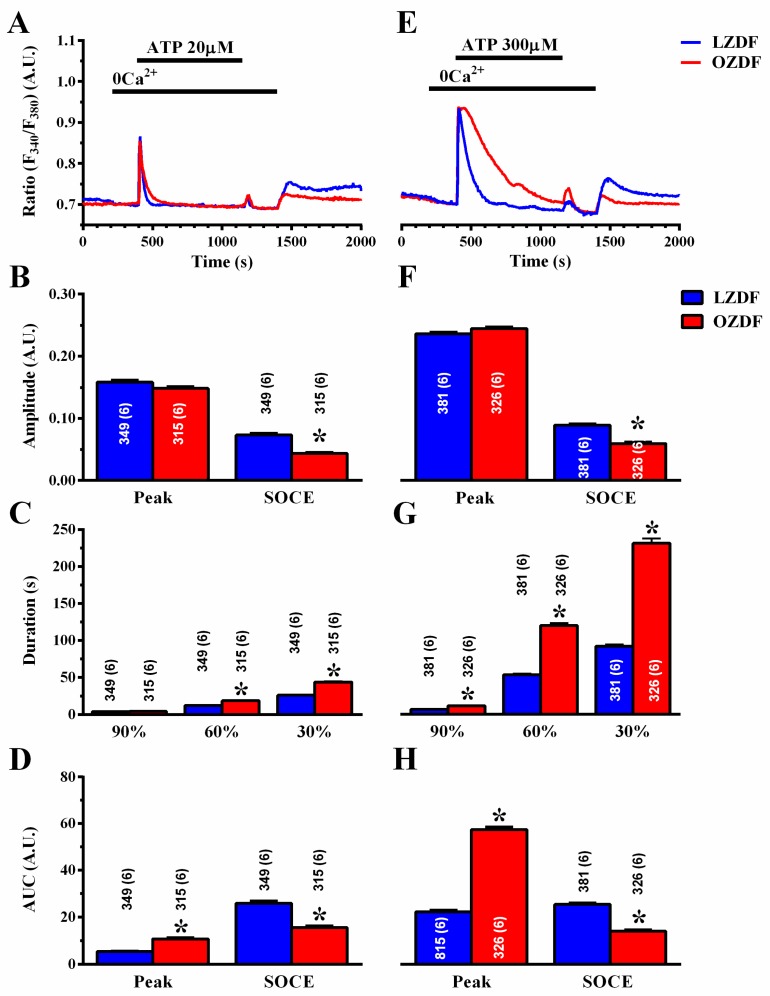
The “Ca^2+^ add-back” protocols reveal subtle alterations in endogenous Ca^2+^ release and SOCE down-regulation in native aortic endothelium of obese Zucker Diabetic Fatty rats. (**A**) Representative recordings of the “Ca^2+^ add-back” protocol obtained from native aortic endothelium of LZDF and OZDF rats challenged with 20 µM ATP in absence of extracellular Ca^2+^ (0Ca^2+^). Subsequently, extracellular Ca^2+^ was returned to the perfusate to activate SOCE. Before SOCE activation, ATP was removed to avoid P_2X_ receptor and second messengers-operated channel activation. (**B**) Mean ± SE of endogenous Ca^2+^ release and SOCE amplitude recorded in native aortic endothelium of LZDF and OZDF rats challenged with 20 µM ATP. The asterisk indicates *p* < 0.05. (**C**) Mean ± SE of the duration of the Ca^2+^ response to 20 µM ATP in LZDF and OZDF rats. The asterisk indicates *p* < 0.05. (**D**) Mean ± SE of the AUC of the two distinct components (i.e., endogenous Ca^2+^ release and SOCE) of the Ca^2+^ response to 20 µM ATP in LZDF and OZDF rats. The asterisk indicates *p* < 0.05. (**E**) Representative recordings of the “Ca^2+^ add-back” protocol obtained from native aortic endothelium of LZDF and OZDF rats challenged with 300 µM ATP in absence of extracellular Ca^2+^ (0Ca^2+^). Subsequently, extracellular Ca^2+^ was returned to the perfusate to activate SOCE. (**F**) Mean ± SE of endogenous Ca^2+^ release and SOCE amplitude recorded in native aortic endothelium of LZDF and OZDF rats challenged with 300 µM ATP. The asterisk indicates *p* < 0.05. (**G**) Mean ± SE of the duration of the Ca^2+^ response to 300 µM ATP in LZDF and OZDF rats. The asterisk indicates *p* < 0.05. (**H**) Mean ± SE of the AUC of the two distinct components (i.e., endogenous Ca^2+^ release and SOCE) of the Ca^2+^ response to 300 µM ATP in LZDF and OZDF rats. The asterisk indicates *p* < 0.05, Mann-Whitney test.

To assess how type 2 diabetes mellitus enhances the Ca^2+^ response to ATP, we employed the “Ca^2+^ add-back” protocol to discriminate between InsP_3_-induced ER Ca^2+^ release and SOCE [20,43]. The intact endothelium of excised rat aorta was first stimulated with 20 µM (Figure 4A) or 300 µM ATP (Figure 4E), in the absence of extracellular Ca^2+^ (0Ca^2+^) to induce InsP_3_-dependent Ca^2+^ release and deplete the ER Ca^2+^ content. Subsequently, extracellular Ca^2+^ was returned to the perfusate to activate SOCE. In both conditions, ATP was removed 250 s before re-addition of extracellular Ca^2+^ to prevent the activation of second messengers-operated channels or ionotropic P_2X_ receptors. As shown in Figure 4B,F left, there was no difference in the amplitude of the intracellular Ca^2+^ peak to 20 or 300 µM ATP, respectively. However, the amplitude of SOCE was significantly (*p* < 0.05) reduced in OZDF as compared to LZDF rats, both in cells stimulated with 20 µM (Figure 4B, right) and in cells stimulated with 300 µM (Figure 4F, right). Likewise, a reduction in the corresponding SOCE AUC was also observed (Figure 4D,H, right). Notably, an increase in the AUC of ATP-evoked endogenous Ca^2+^ release (peak) was observed in OZDF rats at 300 µM (Figure 4H, right) and at 20 µM (Figure 4D, right). As a consequence, the duration of ATP-induced endogenous Ca^2+^ release was significantly enhanced in native endothelial cells from OZDF rats challenged with 300 µM ATP (Figure 4G). Also, the decay of [Ca^2+^]_i_ after the initial Ca^2+^ response (at 60% and 30%) to 20 µM ATP was significantly slowed down in OZDF compared to LZDF rats (Figure 4C).

**Figure 5:**
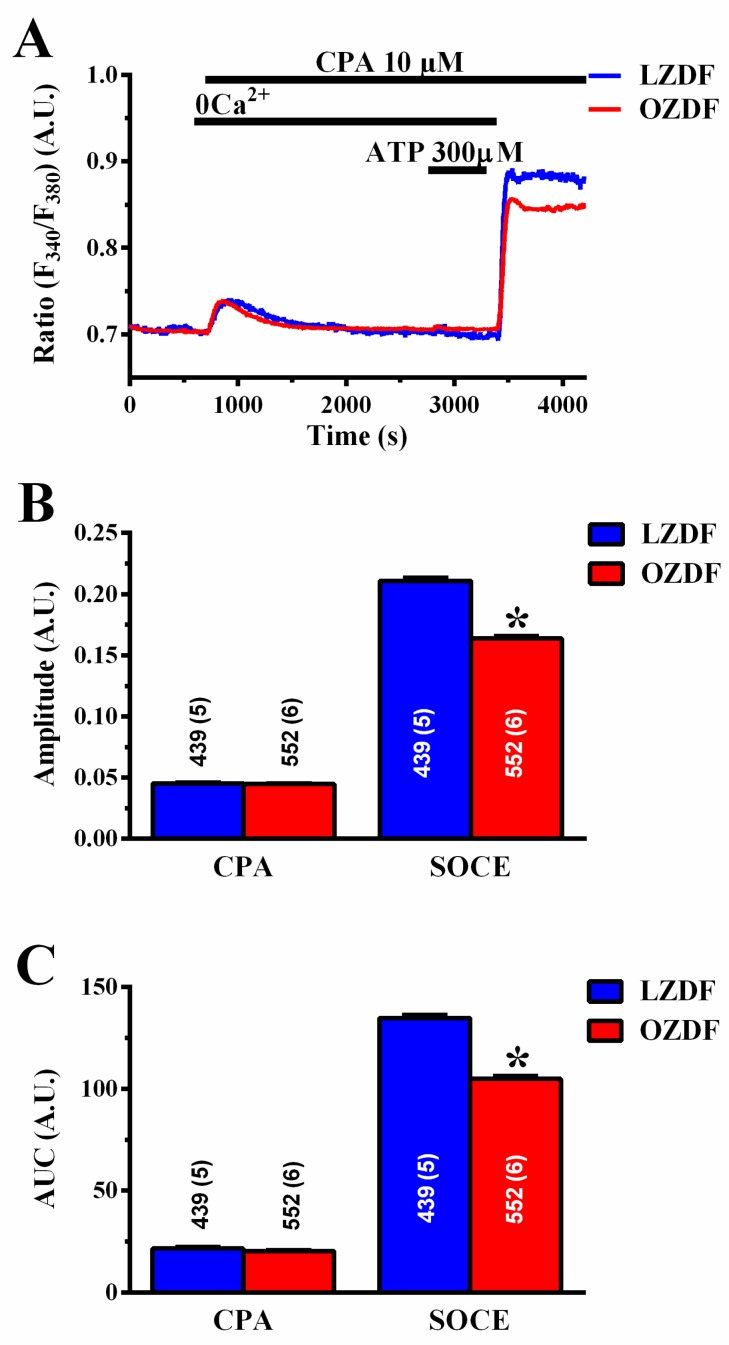
CPA-induced SOCE, but not endogenous Ca^2+^ release, is reduced in native aortic endothelium of obese Zucker diabetic fatty rats. (**A**) Representative recordings of the “Ca^2+^ add-back” protocol obtained from native aortic endothelium of LZDF and OZDF rats challenged with 10 µM CPA. To confirm ER Ca^2+^ pool depletion by CPA, 300 µM ATP was applied in the continuous presence of CPA and in the absence of extracellular Ca^2+^ after recovery of CPA-induced intracellular Ca^2+^ mobilization. (**B**) Mean ± SE of CPA-induced endogenous Ca^2+^ release and SOCE amplitude recorded in native aortic endothelium of LZDF and OZDF rats challenged with 10 µM CPA. (**C**) Mean ± SE of the AUC of the two distinct components (i.e., endogenous Ca^2+^ release and SOCE) of the Ca^2+^ response to 10 µM CPA in LZDF and OZDF rats. The asterisk indicates *p* < 0.05, Mann-Whitney test.

To assess how type 2 diabetes mellitus alters the endothelial Ca^2+^ machinery to rewire the Ca^2+^ response to ATP, we repeated the “Ca^2+^ add-back” protocol by stimulating the intact endothelium of excised rat aorta with cyclopiazonic acid (CPA; 10 µM) [20,36,43]. CPA selectively inhibits SERCA activity, thereby depleting the ER Ca^2+^ pool through yet to be identified ER leak channels and is a widely employed tool to assess ER Ca^2+^ content and SOCE under pathological conditions [27,29,44]. To confirm ER Ca^2+^ pool depletion by CPA, 300 µM ATP was applied in the continuous presence of CPA and in the absence of extracellular Ca^2+^. As observed with ATP, CPA-induced ER Ca^2+^ release was unaltered (Figure 5A–C left), while SOCE was significantly (*p* < 0.05) reduced in OZDF as compared to LZDF rats (Figure 5A–C right). Taken together, these findings strongly suggest that the plateau phase and AUC of the Ca^2+^ response to 20 µM and 300 µM ATP are not enhanced due to an increase in SOCE amplitude. Moreover, it is unlikely that InsP_3_-induced ER Ca^2+^ release is enhanced in OZDF rats. Accordingly, the magnitude of the initial Ca^2+^ response to ATP under 0Ca^2+^ conditions, which is driven by the efflux of intraluminal Ca^2+^ along the electrochemical gradient across ER membrane, is unaltered. Moreover, the amplitude of CPA-induced Ca^2+^ release, which truly reflects the concentration of releasable ER Ca^2+^, is unaffected in OZDF aortae. Endothelial Ca^2+^ signals are finely tuned by the balance between the amount of Ca^2+^ introduced into the cytosol by the opening of Ca^2+^-permeable channels and the amount of Ca^2+^ removed by pumps and transporters on intracellular organelles [21,22,23,24]. Therefore, in the following paragraphs, we seek to elucidate whether and how SERCA, PMCA and NCX contribute to shape the enhanced the Ca^2+^ response induced by 20 and 300 µM ATP in OZDF rats.

### 2.4. The Down-Regulation of SERCA Activity Enhances Plateau Amplitude and Prolongs the Duration of the Ca^2+^ Response to High Doses of ATP in Native Aortic Endothelium of OZDF Rats

**Figure 6:**
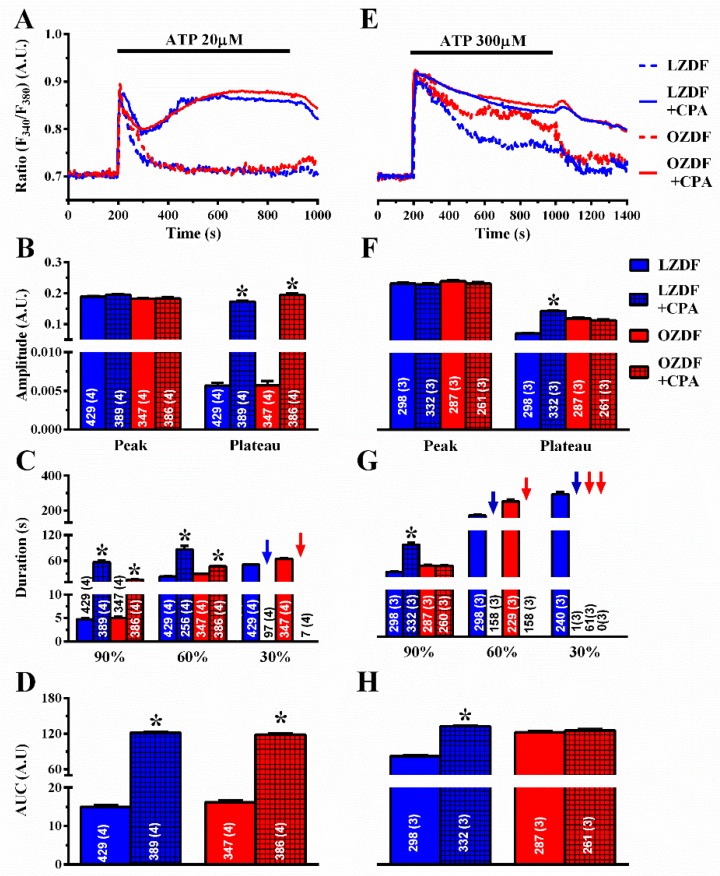
The effect of CPA on the Ca^2+^ response to ATP is larger in native aortic endothelium of obese Zucker diabetic fatty rats. (**A**) Representative recordings of the Ca^2+^ response to 20 µM ATP in the absence or presence of 10 µM CPA in native aortic endothelium of LZDF and OZDF rats. In CPA experiments, ATP and CPA were applied at the same time. (**B**) Mean ± SE of endogenous Ca^2+^ release and SOCE amplitude recorded in native aortic endothelium of LZDF and OZDF rats challenged with 20 µM ATP in the absence or presence of 10 µM CPA. The asterisk indicates *p* < 0.05. (**C**) Mean ± SE of the duration of the two distinct components (i.e., endogenous Ca^2+^ release and SOCE) of the Ca^2+^ response to 20 µM ATP recorded in native aortic endothelium LZDF and OZDF rats challenged with 20 µM ATP in the absence or presence of 10 µM CPA. The asterisk indicates *p* < 0.05. Blue and red arrows indicate that the Ca^2+^ signal failed to reach the clearing rate to the 30% of the initial peak amplitude in LZDF and OZDF rats respectively. (**D**) Mean ± SE of the AUC of the two distinct components (i.e., endogenous Ca^2+^ release and SOCE) of the Ca^2+^ response to 20 µM ATP recorded in native aortic endothelium of LZDF and OZDF rats in the absence or presence of 10 µM CPA. The asterisk indicates *p* < 0.05. (**E**) Representative recordings of the Ca^2+^ response to 300 µM ATP in the absence or presence of 10 µM CPA in native aortic endothelium of LZDF and OZDF rats. In CPA experiments, ATP and CPA were applied at the same time. (**F**) Mean ± SE of endogenous Ca^2+^ release and SOCE amplitude recorded in native aortic endothelium of LZDF and OZDF rats challenged with 300 µM ATP in the absence or presence of 10 µM CPA. The asterisk indicates *p* < 0.05. (**G**) Mean ± SE of the duration of the two distinct components (i.e., endogenous Ca^2+^ release and SOCE) of the Ca^2+^ response to 300 µM ATP in native aortic endothelium of LZDF and OZDF rats in the absence or presence of 10 µM CPA. Blue and red arrows indicate that the Ca^2+^ signal failed to reach the clearing rate indicated in the graph in LZDF and OZDF rats respectively. The asterisk indicates *p* < 0.05. (**H**) Mean ± SE of the AUC of the two distinct components (i.e., endogenous Ca^2+^ release and SOCE) of the Ca^2+^ response to 300 µM ATP in native aortic endothelium of LZDF and OZDF rats in the absence or presence of 10 µM CPA. The asterisk indicates *p* < 0.05, Mann-Whitney test.

To assess whether SERCA affects the Ca^2+^ response to ATP in native aortic endothelium of OZDF, we adopted a strategy previously designed to unravel how it shapes Ca^2+^ signals in rat cardiac microvascular endothelial cells [23]. The Ca^2+^ response to ATP was measured in the absence and in the presence of CPA (10 µM). To ensure that CPA inhibits SERCA activity without altering the ER Ca^2+^ pool, ATP was applied at the same time as CPA [22,23]. As discussed elsewhere [22,23], the CPA- and ATP-sensitive ER Ca^2+^ stores overlap, as also shown in Figure 5A, so that the Ca^2+^ response to CPA does not contaminate the Ca^2+^ response to ATP. The rationale for this experiment is that, if SERCA equally regulates amplitude and kinetics of the plateau phase in OZDF and LZDF, then blocking its activity should affect the Ca^2+^ signal to the same extent in native aortic endothelium of both animals. As shown in Figure 6A, the Ca^2+^ response to 20 µM ATP was dramatically enhanced in the presence of CPA in both LZDF and OZDF rats. The times to 90% and 60% decay of the initial Ca^2+^ peak were significantly slower in the presence of CPA in both animal groups (Figure 6C). Furthermore, CPA blocks SERCA activity to such an extent that the Ca^2+^ signal did not even decay to 30% of the initial amplitude in ≈25% (97 of 389 cells) of LZDF rats and in ≈2% (7 of 386 cells) of OZDF rats (blue and red arrows in Figure 6C), thereby increasing the amplitude of the plateau phase (Figure 6B, right) and the AUC (Figure 6D) to the same extent in OZDF and LZDF rats. Conversely, the blockade of SERCA activity did not alter the amplitude of the initial Ca^2+^ peak (Figure 6B, left).

**Figure 7:**
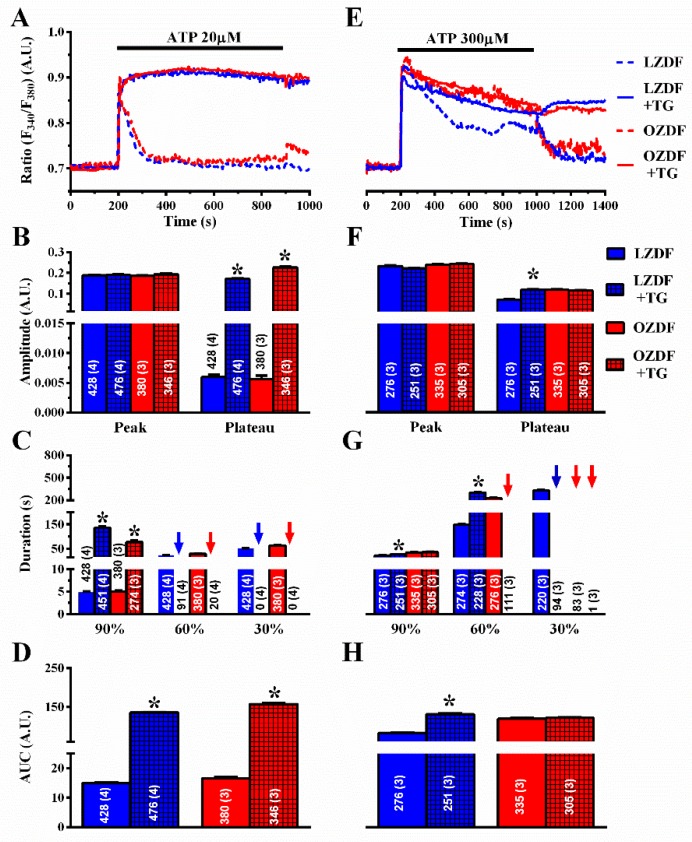
The effect of thapsigargin on the Ca^2+^ response to ATP is larger in native aortic endothelium of obese Zucker diabetic fatty rats. (**A**) Representative recordings of the Ca^2+^ response to 20 µM ATP in the absence or presence of 1 µM thapsigargin (TG in the figure) in native aortic endothelium of LZDF and OZDF rats. In thapsigargin experiments, ATP and thapsigargin were applied at the same time. (**B**) Mean ± SE of endogenous Ca^2+^ release and SOCE amplitude recorded in native aortic endothelium of LZDF and OZDF rats challenged with 20 µM ATP in the absence or presence of 1 µM thapsigargin. The asterisk indicates *p* < 0.05. (**C**) Mean ± SE of the duration of the Ca^2+^ response to 20 µM ATP recorded in native aortic endothelium LZDF and OZDF rats in the absence or presence of 1 µM thapsigargin. The asterisk indicates *p* < 0.05. (**D**) Mean ± SE of the AUC of the Ca^2+^ response to 20 µM ATP recorded in native aortic endothelium of LZDF and OZDF rats in the absence or presence of 1 µM thapsigargin. The asterisk indicates *p* < 0.05. (**E**) Representative recordings of the Ca^2+^ response to 300 µM ATP in the absence or presence of 1 µM thapsigargin in native aortic endothelium of LZDF and OZDF rats. In thapsigargin experiments, ATP and thapsigargin were applied at the same time. (**F**) Mean ± SE of endogenous Ca^2+^ release and SOCE amplitude recorded in native aortic endothelium of LZDF and OZDF rats challenged with 300 µM ATP in the absence or presence of 1 µM thapsigargin. The asterisk indicates *p* < 0.05. (**G**) Mean ± SE of the duration of the Ca^2+^ response to 300 µM ATP in native aortic endothelium of LZDF and OZDF rats in the absence or presence of 1 µM thapsigargin. Blue and red arrows indicate that the Ca^2+^ signal failed to reach the clearing rate indicated in the graph in LZDF and OZDF rats respectively. The asterisk indicates *p* < 0.05. (**H**) Mean ± SE of the AUC of the Ca^2+^ response to 300 µM ATP in native aortic endothelium of LZDF and OZDF rats in the absence or presence of 1 µM thapsigargin. The asterisk indicates *p* < 0.05, Mann-Whitney test.

To confirm this observation, we also exploited thapsigargin (TG) (1 µM), which is another structurally unrelated SERCA inhibitor [23]. As observed with CPA, thapsigargin did not affect the initial Ca^2+^ peak, but dramatically slowed down the early (90%) rate of decay of [Ca^2+^]_i_ in both animal groups (Figure 7A,C). In the presence of thapsigargin, the Ca^2+^ response to 20 µM ATP was maintained at such a high plateau level (Figure 7B, right) that it was not possible to measure the intermediate (60%) clearing rate in ≈19% (91 of 476 cells) of LZDF and ≈6% (20 of 346 cells) of OZDF rats and the late (30%) clearing rate in ≈ 0% (none of 476 cells) of LZDF and ≈ 0% (none of 346 cells) of OZDF rats clearing rates (Figure 7C, blue and red arrows). This led to a remarkable increase in the AUC (Figure 7D) in both animal groups.

However, when the intact rat aorta was stimulated with 300 µM ATP, CPA (Figure 6E–H) and thapsigargin (Figure 7E–H) did not remarkably alter the Ca^2+^ signal in OZDF rats. There was no difference in the amplitude of the initial Ca^2+^ peak (Figure 6F or Figure 7F, left) or of the following plateau (Figure 6F or Figure 7F, right), in the duration of the Ca^2+^ response at 90% of the peak Ca^2+^ amplitude (Figure 6G or Figure 7G), or in the AUC (Figure 6H or Figure 7H) in the absence or in the presence of SERCA inhibition. We only observed an increase in the intermediate (60%) clearing rate such that this value was not measurable in ≈61% (158 of 261 cells) of OZDF rats (Figure 6G or Figure 7G). As expected from Figure 3G, the late (30%) clearing rate was not measurable in either control (≈0.3%; 1 of 332 cells) or treated OZDF rats (0%; none of 261 cells) (Figure 6G or Figure 7G). Conversely, CPA enhanced plateau amplitude (Figure 6F, right), slowed down the duration of the Ca^2+^ signal (Figure 6G), and increased the AUC (Figure 6H) in LZDF rats. Similar effects on LZDF rats were achieved by thapsigargin (Figure 7E–H).

Taken together, these findings strongly suggest that SERCA activity is down-regulated in native endothelium of rat aorta in situ in the presence of type 2 diabetes mellitus. As a consequence, SERCA is not able to cope with the amount of Ca^2+^ introduced into the cytosol when the cells are massively, i.e., at 300 µM, stimulated with ATP. This, in turn, causes the decay rate of [Ca^2+^]_i_ to slow down and increases the plateau phase, although SOCE amplitude is reduced in OZDF rats.

### 2.5. PMCA Activity Is Not Altered in Native Aortic Endothelium of OZDF Rats

**Figure 8:**
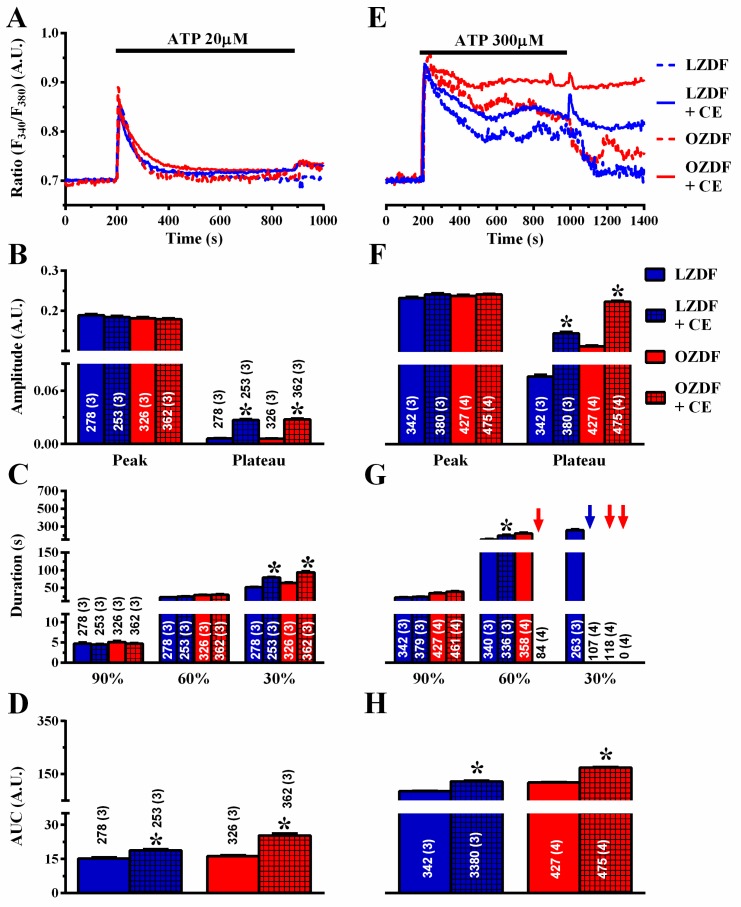
The effect of carboxyeosin on the Ca^2+^ response to ATP is unaltered in native aortic endothelium of obese Zucker diabetic fatty rats. (**A**) Representative recordings of the Ca^2+^ response to 20 µM ATP in the absence or presence of 20 µM carboxyeosin (CE) in native aortic endothelium of LZDF and OZDF rats. Excised rat aorta was preincubated for 500 s with 20 µM CE before ATP addition. (**B**) Mean ± SE of endogenous Ca^2+^ release and SOCE amplitude recorded in native aortic endothelium of LZDF and OZDF rats challenged with 20 µM ATP in the absence or presence of 20 µM CE. (**C**) Mean ± SE of the duration of the Ca^2+^ response to 20 µM ATP recorded in native aortic endothelium of LZDF and OZDF rats in the absence or presence of 20 µM CE. (**D**) Mean ± SE of the AUC of the two distinct components (i.e., endogenous Ca^2+^ release and SOCE) of the Ca^2+^ response to 20 µM ATP recorded in native aortic endothelium of LZDF and OZDF rats in the absence or presence of 20 µM CE. (**E**) Representative recordings of the Ca^2+^ response to 300 µM ATP in the absence or presence of 20 µM CE in native aortic endothelium of LZDF and OZDF rats. (**F**) Mean ± SE of endogenous Ca^2+^ release and SOCE amplitude recorded in native aortic endothelium of LZDF and OZDF rats challenged with 300 µM ATP in the absence or presence of 20 µM CE. The asterisk indicates *p* < 0.05. (**G**) Mean ± SE of the duration of the two distinct components (i.e., endogenous Ca^2+^ release and SOCE) of the Ca^2+^ response to 300 µM ATP in native aortic endothelium of LZDF and OZDF rats in the absence or presence of 20 µM CE. The asterisk indicates *p* < 0.05. Blue and red arrows indicate that the Ca^2+^ signal failed to reach the clearing rate indicated in the graph in LZDF and OZDF rats respectively. (**H**) Mean ± SE of the AUC of the two distinct components (i.e., endogenous Ca^2+^ release and SOCE) of the Ca^2+^ response to 300 µM ATP in native aortic endothelium of LZDF and OZDF rats in the absence or presence of 20 µM CE. The asterisk indicates *p* < 0.05, Mann-Whitney test.

Next, we evaluated whether PMCA activity was impaired in native aortic endothelium of OZDF rats. To address this issue, the Ca^2+^ response to 20 µM and 300 µM ATP was measured in the absence and in the presence of carboxyeosin (CE, 20 µM), a selective PMCA blocker [23]. Carboxyeosin altered the Ca^2+^ signal induced by 20 µM ATP to the same extent in OZDF and LZDF rats (Figure 8A–D): there was no statistically relevant difference in the amplitude of the initial Ca^2+^ peak, while the subsequent plateau phase (Figure 8B), the duration of the Ca^2+^ response at 30% of the transient decay (Figure 8C), and the AUC (Figure 8D) increased in both animal groups. Carboxyeosin also exerted a dramatic impact on the Ca^2+^ signal induced by 300 µM ATP in both OZDF and LZDF rats. A careful analysis of the Ca^2+^ tracings shown in Figure 8E revealed that the [Ca^2+^]_i_ remained high and did not recover to the baseline in either type of rat, so that the late (30%) clearing rate could not be measured in as many as ≈28% (107 of 380 cells) of LZDF rats, although plateau amplitude, late (60 and 30%) decay time, and AUC were still higher in OZDF rats (Figure 8F–H). Taken together, these findings suggest that: 1) PMCA is a major factor in shaping the decay phase and plateau amplitude in response to 20 µM and 300 µM, ATP; and 2) PMCA activity is not remarkably altered by type 2 diabetes mellitus, as its inhibition similarly affects the Ca^2+^ response and maintains the differences in the amplitude and kinetics of the plateau phase observed between OZDF and LZDF rats.

### 2.6. NCX Activity Is Not Altered in Native Aortic Endothelium of OZDF Rats

**Figure 9:**
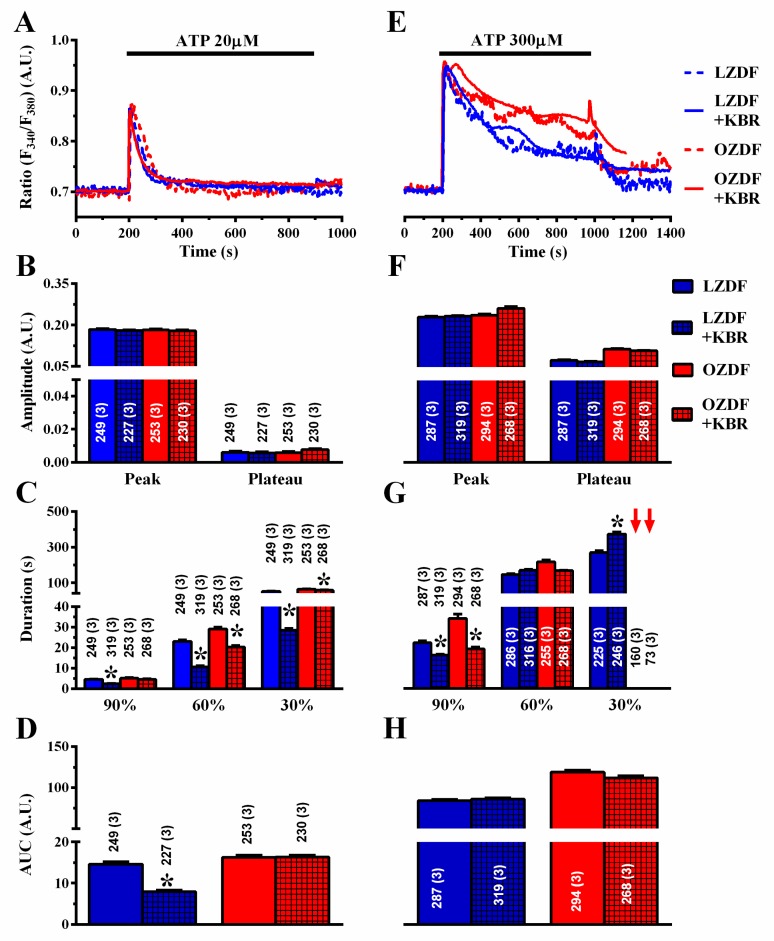
The effect of KB-R7943R on the Ca^2+^ response to ATP is weakly altered in native aortic endothelium of obese Zucker diabetic fatty rats. (**A**) Representative recordings of the Ca^2+^ response to 20 µM ATP in the absence or presence of 8 µM KB-R7943R (KBR) in native aortic endothelium of LZDF and OZDF rats. In KBR experiments, endothelial cells were preincubated for 300 s with 8 µM KBR. (**B**) Mean ± SE of endogenous Ca^2+^ release and SOCE amplitude recorded in native aortic endothelium of LZDF and OZDF rats challenged with 20 µM ATP in the absence or presence of 8 µM KBR. (**C**) Mean ± SE of the duration of the Ca^2+^ response to 20 µM ATP recorded in native aortic endothelium of LZDF and OZDF rats in the absence or presence of 8 µM KBR. (**D**) Mean ± SE of the AUC of the two distinct components (i.e., endogenous Ca^2+^ release and SOCE) of the Ca^2+^ response to 20 µM ATP recorded in native aortic endothelium of LZDF and OZDF rats in the absence or presence of 8 µM KBR. (**E**) Representative recordings of the Ca^2+^ response to 300 µM ATP in the absence or presence of 8 µM KBR in native aortic endothelium of LZDF and OZDF rats. (**F**) Mean ± SE of endogenous Ca^2+^ release and SOCE amplitude recorded in native aortic endothelium of LZDF and OZDF rats challenged with 300 µM ATP in the absence or presence of 8 µM KBR. The asterisk indicates *p* < 0.05. (**G**) Mean ± SE of the duration of the two distinct components (i.e., endogenous Ca^2+^ release and SOCE) of the Ca^2+^ response to 300 µM ATP in native aortic endothelium of LZDF and OZDF rats in the absence or presence of 8 µM KBR. The asterisk indicates *p* < 0.05. Red arrows indicate that the Ca^2+^ signal failed to reach the clearing rate indicated in the graph in OZDF rats. (**H**) Mean ± SE of the AUC of the two distinct components (i.e., endogenous Ca^2+^ release and SOCE) of the Ca^2+^ response to 300 µM ATP in native aortic endothelium of LZDF and OZDF rats in the absence or presence of 8 µM KBR. The asterisk indicates *p* < 0.05, Mann-Whitney test.

**Figure 10:**
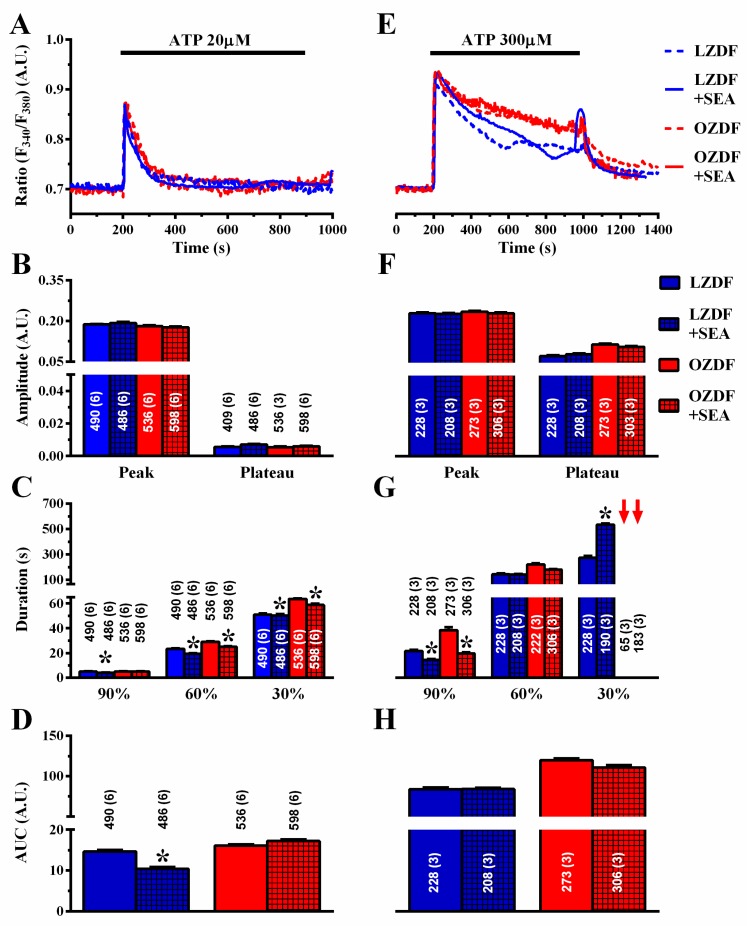
The effect of SEA0400 on the Ca^2+^ response to ATP is weakly altered in native aortic endothelium of obese Zucker diabetic fatty rats. (**A**) Representative recordings of the Ca^2+^ response to 20 µM ATP in the absence or presence of 3 µM SEA0400 (SEA) in native aortic endothelium of LZDF and OZDF rats. In SEA experiments, endothelial cells were preincubated for 600 s with 3 µM SEA. (**B**) Mean ± SE of endogenous Ca^2+^ release and SOCE amplitude recorded in native aortic endothelium of LZDF and OZDF rats challenged with 20 µM ATP in the absence or presence of 3 µM SEA. (**C**) Mean ± SE of the duration of the two distinct components (i.e., endogenous Ca^2+^ release and SOCE) of the Ca^2+^ response to 20 µM ATP in native aortic endothelium of LZDF and OZDF rats in the absence or presence of 3 µM SEA. Red arrows indicate that the Ca^2+^ signal failed to reach the clearing rate in OZDF rats. (**D**) Mean ± SE of the AUC of the two distinct components (i.e., endogenous Ca^2+^ release and SOCE) of the Ca^2+^ response to 20 µM ATP in native aortic endothelium of LZDF and OZDF rats in the absence or presence of 3 µM SEA. (**E**) Representative recordings of the Ca^2+^ response to 300 µM ATP in the absence or presence of 3 µM SEA0400 (SEA) in native aortic endothelium of LZDF and OZDF rats. In SEA experiments, endothelial cells were preincubated for 600s with 3 µM SEA. (**F**) Mean ± SE of endogenous Ca^2+^ release and SOCE amplitude recorded in native aortic endothelium of LZDF and OZDF rats challenged with 300 µM ATP in the absence or presence of 3 µM SEA. (**G**) Mean ± SE of the duration of the two distinct components (i.e., endogenous Ca^2+^ release and SOCE) of the Ca^2+^ response to 300 µM ATP in native aortic endothelium of LZDF and OZDF rats in the absence or presence of 3 µM SEA. Red arrows indicate that the Ca^2+^ signal failed to reach the clearing rate in OZDF rats. (**H**) Mean ± SE of the AUC of the two distinct components (i.e., endogenous Ca^2+^ release and SOCE) of the Ca^2+^ response to 300 µM ATP in native aortic endothelium of LZDF and OZDF rats in the absence or presence of 3 µM SEA. The asterisk indicates *p* < 0.05, Mann-Whitney test.

Finally, we assessed the role of NCX in native aortic endothelium of OZDF and LZDF rats by first measuring the Ca^2+^ response to ATP in the presence and in the absence of KBR-79433 (KBR), a selective inhibitor of NCX activity [21]. When the intact aorta was stimulated with 20 µM ATP (Figure 9A), the peak and plateau amplitude (Figure 9B) were not statistically different in either LZDF or OZDF rats. However, there was a significant (*p* < 0.05) reduction in the clearing rate at 90%, 60% and 30% of the initial Ca^2+^ peak (Figure 9C) and in the AUC (Figure 9D) in LZDF rats. Conversely, no statistically relevant difference was found in the early (90%) decay time (Figure 8C) and in the AUC (Figure 9D) in OZDF rats. The effect of KBR (8 µM) was slightly different when rat aortae were challenged with 300 µM ATP (Figure 9E–H). We only observed a significant (*p* < 0.05) reduction in the 90% decay times and an increase in the 30% clearing rate in both LZDF and OZDF rats (Figure 9G). However, there was no significant change in the initial Ca^2+^ peak (Figure 9F), in the plateau amplitude (Figure 9F), or in the AUC (Figure 9H). The same findings were obtained when the Ca^2+^ signal induced by 300 µM ATP was measured in the presence of SEA0400 (SEA) (3 µM), which blocks both the forward and reverse mode of NCX [45] (Figure 10). Overall, these results demonstrate that NCX negatively regulates intracellular Ca^2+^ clearing when rat aortic endothelium is stimulated with low (i.e., 20 µM) ATP concentration, although this effect significantly reduces the AUC only in LZDF rats. However, NCX inhibition does not affect the AUC or the initial peak and plateau phase of the Ca^2+^ response at high (e.g., 300 µM)-intensity ATP stimulation in either LZDF or OZDF rats.

### 2.7. SERCA2B Protein Is Up-Regulated in in Native Aortic Endothelium of OZDF Rats

**Figure 11:**
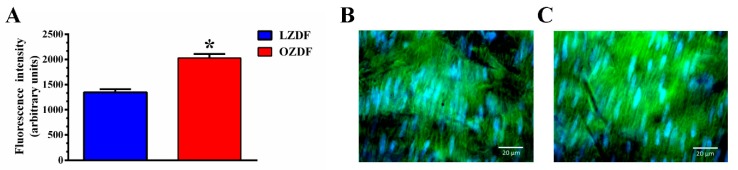
SERCA2B protein is upregulated in native aortic endothelium of obese Zucker rats. Semiquantitative analysis (**A**) of immunofluorescent staining of the endothelial monolayer of rat aorta revealed that SERC2B expression was up-regulated in OZDF (**C**) as compared to LZDF (**B**) rats (SERCA2B: red; blue: cell nuclei). The asterisk indicates *p* < 0.05.

The pharmacological manipulation of the Ca^2+^ response to low (i.e., 20 µM) and high (i.e., 300 µM) doses of ATP strongly suggested that SERCA-dependent Ca^2+^ removal from the cytosol is compromised in in situ aorta of OZDF rats. The inhibition of SERCA-dependent Ca^2+^ clearance could be due to the down-regulation of SERCA2B, the main endothelial SERCA isoform [46], and/or to the slowing down of its activity. Previous work has revealed that, while SERCA2 protein is up-regulated by type 2 diabetes mellitus, its clearing rate may be severely compromised. Accordingly, immunohistochemical analysis carried out with a specific antibody revealed that the SERCA2 expression was significantly (*p* < 0.05) enhanced in native aortic endothelium of OZDF as compared to LZDF rats (Figure 11). As illustrated in Figure 11B,C and summarized in Figure 11A, the endothelial SERCA2B staining was brighter in in situ rat aorta from OZDF rats. This finding therefore indicates that endothelial SERCA2B activity is inhibited by type 2 diabetes mellitus.

### 2.8. ROS Inhibition Rescues SERCA2B-Dependent Ca^2+^ Sequestration in Native Aortic Endothelium of OZDF Rats

**Figure 12:**
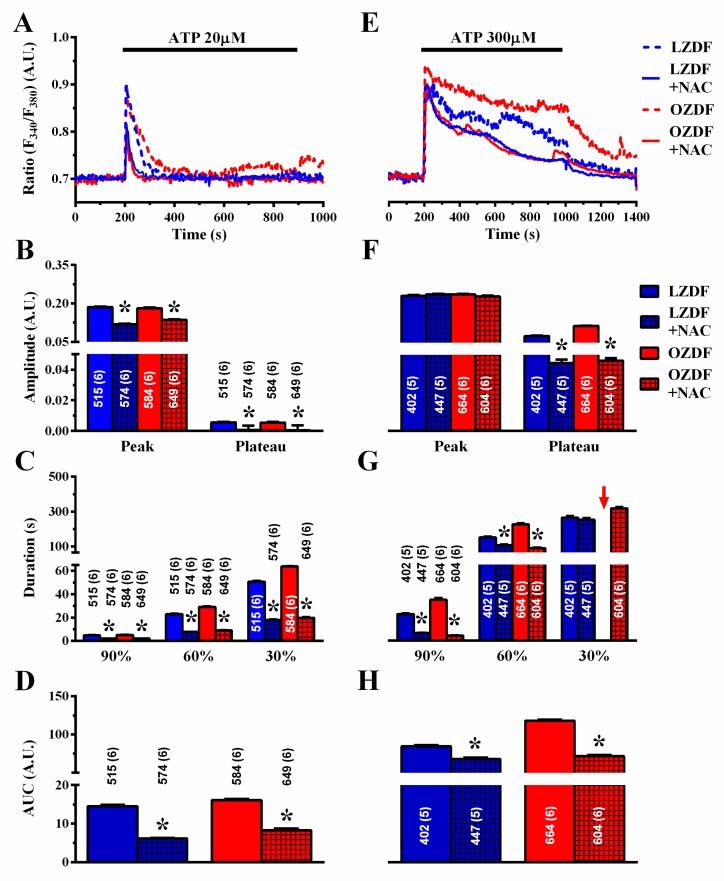
Scavenging ROS with NAC tempers the differences in the Ca^2+^ response to ATP in obese Zucker diabetic fatty rats. (**A**) Representative recordings of the Ca^2+^ response to 20 µM ATP in the absence or presence of 3 mM NAC in native aortic endothelium of LZDF and OZDF rats. Rat aortic rings were preincubated for 1 h with 3 mM NAC. (**B**) Mean ± SE of endogenous Ca^2+^ release and SOCE amplitude recorded in native aortic endothelium of LZDF and OZDF rats challenged with 20 µM ATP in the absence or presence of 3 mM NAC. (**C**) Mean ± SE of the duration of the Ca^2+^ response to 20 µM ATP recorded in native aortic endothelium of LZDF and OZDF rats in the absence or presence of 3 mM NAC. (**D**) Mean ± SE of the AUC of the two distinct components (i.e., endogenous Ca^2+^ release and SOCE) of the Ca^2+^ response to 20 µM ATP recorded in native aortic endothelium of LZDF and OZDF rats in the absence or presence of 3 mM µM NAC. (**E**) Representative recordings of the Ca^2+^ response to 300 µM ATP in the absence or presence of 3 mM NAC in native aortic endothelium of LZDF and OZDF rats. (**F**) Mean ± SE of endogenous Ca^2+^ release and SOCE amplitude recorded in native aortic endothelium of LZDF and OZDF rats challenged with 300 µM ATP in the absence or presence of 3 mM NAC. The asterisk indicates *p* < 0.05. (**G**) Mean ± SE of the duration of the two distinct components (i.e., endogenous Ca^2+^ release and SOCE) of the Ca^2+^ response to 300 µM ATP in native aortic endothelium of LZDF and OZDF rats in the absence or presence of 3 mM µM NAC. The asterisk indicates *p* < 0.05. Red arrows indicated that the Ca^2+^ signal failed to reach the clearing rate indicated in the graph in OZDF rats. (**H**) Mean ± SE of the AUC of the two distinct components (i.e., endogenous Ca^2+^ release and SOCE) of the Ca^2+^ response to 300 µM ATP in native aortic endothelium of LZDF and OZDF rats in the absence or presence of 3 mM NAC. The asterisk indicates *p* < 0.05, Mann-Whitney test.

Several reports have demonstrated that SERCA activity could be inhibited by reactive oxygen species (ROS) under hyperglycemic conditions [47] and in prediabetic Zucker rats [48]. Furthermore, vascular ROS production is enhanced in OZDF rats [49]. We reasoned that, if SERCA2B inhibition depends on enhanced oxidative stress, then the ROS scavenger N-acetyl-L-cysteine (NAC) should reduce the duration and the plateau phase of the Ca^2+^ response to ATP in native aortic endothelium of OZDF rats. As shown in Figure 12A, NAC (3 mM, 1 h incubation) also impacted on the Ca^2+^ signal induced by 20 µM ATP. The amplitude of the Ca^2+^ peak and of the plateau phase (Figure 12B), the duration (Figure 12C), and AUC (Figure 12D) were significantly reduced by pre-treating rat aortic rings with NAC. As is evident from the Ca^2+^ traces shown in Figure 12A and from the statistical analysis of each parameter (Figure 12B–D), there was no longer a difference between the Ca^2+^ signals induced by 20 µM ATP in LZDF vs. OZDF rats. Furthermore, scavenging ROS with NAC eliminated the differences in the amplitude and kinetics of the Ca^2+^ responses to 300 µM ATP (Figure 12E). Statistical analysis revealed that following pre-treatment with NAC, there was no statistically relevant difference in plateau amplitude (Figure 12F), duration (Figure 12G), or AUC (Figure 12H) between LZDF and OZDF rats. These data, therefore, strongly suggest that the down-regulation of SERCA2B activity by the enhanced oxidative stress imposed to OZDF rats is responsible for the dismantling of endothelial Ca^2+^ dynamics in rat aorta.

### 2.9. Evidence for the Contribution of K^+^ Channels in the Alteration of Intracellular Ca^2+^ Dynamics in Native Aortic Endothelium of OZDF Rats

**Figure 13:**
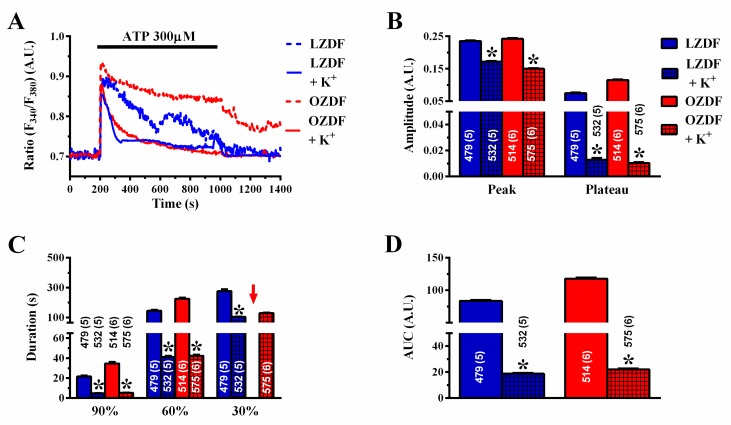
High extracellular KCl affects the Ca^2+^ response to ATP in obese Zucker diabetic fatty rats. (**A**) Representative recordings of the Ca^2+^ response to 300 µM ATP in the absence or presence of high-KCl (90 mM) extracellular solution in native aortic endothelium of LZDF and OZDF rats. (**B**) Mean ± SE of endogenous Ca^2+^ release and SOCE amplitude recorded in native aortic endothelium of LZDF and OZDF rats challenged with 300 µM ATP in the absence or presence of high-KCl extracellular solution. The asterisk indicates *p* < 0.05. (**C**) Mean ± SE of the duration of the two distinct components (i.e., endogenous Ca^2+^ release and SOCE) of the Ca^2+^ response to 300 µM ATP in native aortic endothelium of LZDF and OZDF rats in the absence or presence of high-KCl extracellular solution. The asterisk indicates *p* < 0.05. Red arrows indicated that the Ca^2+^ signal failed to reach the clearing rate indicated in the graph in OZDF rats. (**D**) Mean ± SE of the AUC of the two distinct components (i.e., endogenous Ca^2+^ release and SOCE) of the Ca^2+^ response to 300 µM ATP in native aortic endothelium of LZDF and OZDF rats in the absence or presence of high-KCl extracellular solution. The asterisk indicates *p* < 0.05, Mann-Whitney test.

It has been demonstrated that type 2 diabetes mellitus may also effect the expression of intermediate- and small-conductance Ca^2+^-dependent K^+^ (IK_Ca_ and SK_Ca_, respectively) channels, which can be recruited by an increase in [Ca^2+^]_i_, thereby increasing the driving force for extracellular Ca^2+^ entry [50]. As SOCE amplitude is down-regulated in native aortic endothelium of OZDF rats despite the fact that endothelial hyperpolarization is likely to be increased, abrogating the electrochemical gradient of K^+^ across the plasma membrane should further decrease ATP-induced Ca^2+^ entry. If this hypothesis is correct, then we should observe a reduction in the plateau phase that the slowing down of SERCA2B activity might not be able to counteract. As expected, when rat aortae were challenged with 300 µM ATP in the presence of high extracellular K^+^ (90 mM) (Figure 13A), the magnitude of the plateau phase (Figure 13B), the duration of the Ca^2+^ signal (Figure 13C), and the AUC (Figure 13D) were significantly (*p* < 0.05) reduced in both LZDF and OZDF rats. Of note, the amplitude and kinetics of the Ca^2+^ traces were no longer different, as clearly shown in Figure 13A. This finding suggests that the further decrease in extracellular Ca^2+^ entry in high-KCl extracellular solution, when associated with the slowing down of SERCA2B-mediated Ca^2+^ removal from the cytosol in OZDF rats, results in a Ca^2+^ signal that is not different from that recorded in lean animals.

## 3. Discussion

The present investigation demonstrated for the first time that the Ca^2+^ handling machinery is dramatically altered in native endothelium of excised aorta of OZDF rats, which represent a widespread model of type 2 diabetes mellitus. Remodeling of the Ca^2+^ signaling toolkit was fully disclosed by stimulating native endothelial cells with high doses, i.e., 300 µM, of ATP and consisted of a remarkable increase in the duration of the Ca^2+^ signal and in plateau amplitude. Dysregulation of the endothelial Ca^2+^ machinery was also detectable, although at a lower extent (i.e., duration of the Ca^2+^ signal), at low ATP doses, i.e., 20 µM. These findings might shed new light on the molecular mechanisms by which type 2 diabetes mellitus causes endothelial dysfunction, thereby severely affecting the cardiovascular system and compromising patients’ health [4,5,7,8].

Earlier studies revealed that high glucose may enhance endothelial SOCE by up-regulating its molecular components [51,52], namely Stim1 and Orai1, which serve as the ER Ca^2+^ sensor and the pore-forming subunit of the Ca^2+^-permeable channel, respectively [19,53]. Furthermore, a more recent investigation disclosed that SERCA activity is compromised in a rat model of type 1 diabetes mellitus [54]. Nevertheless, no study has been devoted to investigating whether the Ca^2+^ handling machinery is impaired in vascular endothelial cells in the presence of type 2 diabetes mellitus. Therefore, we focused on intact aortic endothelium of OZDF rats, which enabled us to investigate intracellular Ca^2+^ dynamics in a cellular model which reflects the physiological configuration of vascular endothelial cells in type 2 diabetes mellitus. We provided the evidence that: (1) the mechanisms responsible for clearing cytosolic Ca^2+^ in LZDF rats, which represent the lean age-matched control, slightly differ depending on ATP concentration ([ATP]); (2) the Ca^2+^-transporting system is affected in intact aortic endothelium of OZDF rats; and (3) this impairment is fully unmasked by stimulating the cells with the highest ATP dose, i.e., 300 µM. Therefore, we will first describe how SERCA, PMCA and NCX interact to remove cytosolic Ca^2+^ under physiological conditions, i.e., in native endothelium of excised aorta of LZDF rats, and then discuss the remodeling of the Ca^2+^ handling machinery in OZDF rats.

### 3.1. Ca^2+^ Clearing in LZDF Rats: the Control Condition

ATP induces a biphasic Ca^2+^ response in native rat aortic endothelium which consists in an initial Ca^2+^ peak, due to InsP_3_-dependent ER Ca^2+^ release, followed by SOCE activation [36,39]. Herein, we first showed that ATP elicits a dose-dependent increase in the amplitude of the initial Ca^2+^ peak and in SOCE amplitude, as depicted in Figure 3. We further demonstrated that SERCA is a major mechanism responsible for clearing cytosolic Ca^2+^ in response to weak, i.e., 20 µM, and massive, i.e., 300 µM, stimulation with ATP. This conclusion is supported by the observation that blocking SERCA activity with CPA and thapsigargin prevents [Ca^2+^]_i_ from declining to the baseline, thereby enhancing the plateau phase and prolonging the duration of the Ca^2+^ response, which collectively lead to an increase in the AUC. CPA is also known to stimulate ER Ca^2+^ release and activate SOCE in intact aortic endothelium [36]. However, CPA was administered at the same time as ATP, a procedure which enabled this drug to inhibit SERCA activity without altering the ER Ca^2+^ pool [22,23]. Moreover, the InsP_3_-sensitive store targeted by ATP overlaps with the ER Ca^2+^ content, so that both stimuli converge on the recruitment of the same SOCE pathway [24,36]. Therefore, the dramatic increase in plateau amplitude observed in the presence of CPA is not due to further SOCE activation, but to the impairment of Ca^2+^ sequestration into ER lumen, which results in Ca^2+^ accumulation within the cytosol. It has been shown that ER Ca^2+^ refilling occurs at plasma membrane/ER nanojunctions [55,56], which recruit SERCA upon massive ER Ca^2+^ depletion [57,58], in vascular endothelial cells. Accordingly, InsP_3_-induced drop in ER Ca^2+^ concentration causes SERCA migration and co-localization with Stim1 and Orai1, thereby resulting in more efficient Ca^2+^ pumping into the ER [57,58]. Therefore, we hypothesize that SERCA is heavily recruited to plasma membrane/ER nanojunctions following ATP stimulation and SOCE activation.

In addition to SERCA, PMCA intervenes to remove cytosolic Ca^2+^ in response to 20 µM and 300 µM ATP. Accordingly, blocking PMCA activity with carboxyeosin enhanced plateau amplitude and slowed down the decay phase of the Ca^2+^ in response to both low, i.e., 20 µM, and high, i.e., 300 µM, ATP doses. As a consequence, the AUC also underwent a remarkable increase. In agreement with these observations, earlier work showed that endothelial SOCE is functionally associated with PMCA, which is activated by incoming Ca^2+^ and supports SERCA activity during the decline of [Ca^2+^]_i_ [59,60].

The contribution of NCX to the endothelial Ca^2+^ response to ATP is more puzzling to decipher. The blockade of NCX activity with two structurally distinct drugs, i.e., KBR and SEA, decreased the duration of the Ca^2+^ response to 20 µM ATP, thereby causing a significant reduction in the AUC. This effect was detected in the absence of extracellular Ca^2+^ and cannot be ascribed to the inhibition of the reverse-mode NCX activity and is unlikely to reflect off-target effects. It has, however, long been known that NCX and SERCA compete for clearing Ca^2+^ during the decay phase of a Ca^2+^ transient and that the clearing rate of SERCA activity may be remarkably faster as compared to NCX [61]. Therefore, we posit that, upon NCX inhibition, SERCA is responsible for clearing most of the Ca^2+^ released by 20 µM ATP and for the subsequent acceleration of the decay phase. Notably, the blockade of NCX activity exerts a weaker impact on the Ca^2+^ response to high, i.e., 300 µM, ATP doses, as the AUC is not affected, and only the early (90%) clearing rate is shortened. This observation strongly suggests that NCX plays a minor role in shaping the Ca^2+^ signal induced by massive ATP stimulation, which is in agreement with the notion that higher SOCE activation is likely to result in SERCA recruitment to ER/PM nanojunctions and PMCA up-regulation (see above). Conversely, NCX significantly cooperate with SERCA to clear cytosolic Ca^2+^ in freshly isolated rabbit aortic endothelial cells [24] and cardiac microvascular endothelial cells [11].

### 3.2. Evidence that SERCA2B Activity Is Slowed Down in Native Aortic Endothelium of LZDF Rats

The Ca^2+^ handling machinery that shapes the Ca^2+^ response to ATP is remodeled in the intact endothelium of excised aorta of OZDF rats mainly due to the impairment of SERCA2B activity. The following pieces of evidence support this hypothesis. First, SOCE evoked by ER Ca^2+^ depletion with 20 µM and 300 µM ATP and with CPA is lower in the native endothelium of excised rat aorta of OZDF as compared to LZDF rat, while there is seemingly no difference in the InsP_3_-releasable ER Ca^2+^ pool. Nevertheless, the plateau phase and duration of the Ca^2+^ signal induced by 20 µM and 300 µM ATP are significantly higher in diabetic rats. Therefore, the larger Ca^2+^ signal observed in OZDF rats is not due to the rewiring of Ca^2+^ entry/release channels. Second, the Ca^2+^ response to 20 µM ATP is similarly affected by pharmacological blockade of SERCA2B activity with CPA or thapsigargin in LZDF and OZDF rats: the decay rate was lengthened, while the plateau amplitude and AUC increased. Nevertheless, a careful analysis revealed that the early (90%) and intermediate (60%) clearing rates were significantly (*p* < 0.05) more slowed down in LZDF rather than OZDF rats. This observation suggests that SERCA activity is slightly faster in the native aortic endothelium of LZDF rats. Coherently, the effect of CPA and thapsigargin on the plateau amplitude, duration and AUC of the Ca^2+^ response to 300 µM ATP are larger in the intact aorta of LZDF rather than OZDF rats. This finding concurs with the hypothesis that SERCA activity undergoes a partial inhibition in native aortic endothelium of OZDF rats, which is fully unmasked at high ATP concentration ([ATP]) and dampens the inhibitory effect of CPA and thapsigargin. Third, blocking PMCA activity with carboxyeosin exerted a similar impact on the decay phase of the Ca^2+^ signal induced by 20 µM and 300 µM ATP in LZDF and OZDF rats (i.e., elongation of the decay phase, increase in the plateau amplitude and AUC). This observation indicates that endothelial PMCA activity is not altered by type 2 diabetes mellitus. Fourth, NCX inhibition with KBR or SEA only weakly affected the Ca^2+^ response to 20 µM ATP (i.e., no effect on the AUC, although the clearing rate was slowed down) in OZDF rats, while it exerted a similar effect on the Ca^2+^ signal evoked by 300 µM ATP (i.e., elongation of the decay phase) in LZDF and OZDF rats.

It should also be pointed out that the amplitude, AUC and duration of the CPA-endogenous Ca^2+^ release are not different between LZFD and OZDF aortae. This finding indicates that: (1) the releasable ER Ca^2+^ content; and (2) NCX and PMCX activities, which are the major Ca^2+^-clearing mechanisms acting under such conditions, are not heavily altered in the intact endothelium of OZDF rats. Taken together, these observations strongly suggest that SERCA is the major Ca^2+^-transporting mechanism to be affected in native endothelium of excised aorta from OZDF rats. When the cells are stimulated with a low [ATP], i.e., 20 µM ATP, SERCA2B is still able to cope with the amount of Ca^2+^ introduced into the cytosol by InsP_3_-induced Ca^2+^ release and SOCE in both LZDF and OZDF rats. However, its impaired activity causes [Ca^2+^]_i_ to decay slower to the baseline, thereby slightly enhancing the AUC of the Ca^2+^ signal. When ATP concentration is increased up to 300 µM, the higher amount of Ca^2+^ delivered into the cytosol of OZDF aortae through InsP_3_ receptors and SOCE results in an excessive influx of Ca^2+^ that cannot be entirely sequestered into ER lumen by the impaired SERCA2B and is mainly extruded by PMCA.

The slowing down of endothelial SERCA2B activity in OZDF aortae could be due to the down-regulation of SERCA2B expression and/or to the impairment of its cycling rate. SERCA2B activity is impaired in both type 1 and type 2 diabetes mellitus [47]. Furthermore, while myocardial SERCA 2A expression increased in OZDF rats [62], SERCA2B levels were unchanged in cardiac endothelial cells from a rat model of type 1 diabetes mellitus, although SERCA2B-mediated Ca^2+^ sequestration was significantly dampened [54]. Immunohistochemistry revealed that SERCA2B expression was up-, rather than down-, regulated in native aortic endothelium of OZDF rats. This finding, therefore, strongly suggests that endothelial SERCA2B activity is compromised by type 2 diabetes mellitus. Several mechanisms have been put forward to describe the impairment of SERCA2B-mediated ER Ca^2+^ sequestration into ER lumen in type 2 diabetes mellitus, including oxidative stress and Ca^2+^-ATPase glycation [47]. For instance, a recent investigation disclosed that SERCA2B activity was inhibited by irreversible oxidation of cysteine-647 in OZDF vascular smooth muscle cells. SERCA2B oxidation, in turn, requires NADPH oxidase 4 (Nox4) up-regulation Nox4 by transforming growth factor-β1 [48]. Earlier reports indicated that vascular ROS production was exacerbated in OZDF rats, thereby impairing endothelium-dependent NO release and vasodilation [49]. In addition, oxidative stress was also increased in the endothelial glomerulal layer [63] and coronary microvascular endothelial cells [64] of OZDF rats. Notably, the ROS scavenger NAC abolished the differences in the Ca^2+^ signal elicited by ATP at both 20 µM and 300 µM. First, it should be noted that NAC erased the small plateau phase, which followed 20 µM ATP-induced intracellular Ca^2+^ release, thereby turning a biphasic Ca^2+^ signal into a transient Ca^2+^ increase. This finding suggests that ROS are also able to modulate SERCA2B activity in the endothelial monolayer of lean animals, although their impact on intracellular Ca^2+^ homeostasis becomes more relevant in diabetic rats. Therefore, the low amplitude plateau phase arising in response to low, i.e., 20 µM, doses of ATP depends on partial SERCA2B inhibition rather than on SOCE activation in native endothelium of rat aorta [39]. Second, NAC pretreatment erased any significant difference in the amplitude and kinetics of the Ca^2+^ response to 300 µM ATP between LZDF and OZDF rats. The inhibitory effects on SOCE amplitude on the decay rate (which was accelerated) and on the AUC were stronger in OZDF rats. These observations support the hypothesis that enhanced ROS production dramatically inhibits SERCA2B-dependent Ca^2+^ clearance when the amount of Ca^2+^ introduced in the cytosol through InsP_3_Rs is increased by the stimulation with higher doses of the agonist. It should, however, be recalled that the role of mitochondria in this context remains to be elucidated. Recent investigations revealed that mitochondrial Ca^2+^ accumulation was compromised in mouse hearts, while it was up-regulated in rat liver [65] in the presence of type 1 diabetes mellitus [66]. Assessing whether and how mitochondrial-dependent modulation of intracellular Ca^2+^ signaling is altered in native aortic endothelium of OZDF rats will require the exploration of multiple aspects of endothelial Ca^2+^ signaling. Previous work has indeed shown that, while mitochondria barely control global InsP_3_-dependent ER Ca^2+^ release in vascular endothelial cells [23,43], they finely regulate local InsP_3_-dependent Ca^2+^ signals [67], SOCE amplitude [68], and ER refilling [69].

### 3.3. Preliminary Evidence that Ca^2+^-Dependent K^+^ Channels Contribute to the Enhanced Ca^2+^ Response to ATP

The evidence reported so far clearly has demonstrated that ROS-dependent inhibition of SERCA2B activity exacerbates the Ca^2+^ response to low and, more remarkably, to high doses of ATP. Nevertheless, it has long been known that type 2 diabetes mellitus may also enhance agonist-induced endothelial hyperpolarization by increasing the expression of IK_Ca_ and/or SK_Ca_ channels [50]. For instance, acetylcholine-induced vasodilation was preserved in coronary arteries despite reduced NO bioavailability because of IK_Ca_ and SK_Ca_ up-regulation [70]. Likewise, IK_Ca_ was up-regulated and compensated for lower NO release OZDF mesenteric arteries stimulated with acetylcholine [71]. As discussed elsewhere [72], endothelial hyperpolarization enhances the driving force for agonist-induced extracellular Ca^2+^ entry in vascular endothelium. We reasoned that, if agonist-induced endothelial hyperpolarization modulated extracellular Ca^2+^ entry, then abrogating K^+^ fluxes would exert a major effect in OZDF rats, where it is higher. In agreement with this hypothesis, increasing the extracellular K^+^ concentration to prevent endothelial hyperpolarization had a stronger impact in native aortic endothelium of OZDF rats (see Figure 13A). A careful inspection of the Ca^2+^ tracings and statistical analysis revealed that the Ca^2+^ response to 300 µM ATP did not differ in the presence of high KCl solution between LZDF and OZDF rats. As discussed above, SOCE, which represents the major mechanism for ATP-induced Ca^2+^ influx in rat aortic endothelium, is down-regulated in OZDF rats. Nevertheless, the ROS-dependent inhibition of SERCA2B results in an enhancement of the Ca^2+^ response to ATP. However, a further decrease in extracellular Ca^2+^ entry following reduction of the driving force for Ca^2+^ entry in the presence of high extracellular KCl, may lead to a lower increase in [Ca^2+^]_i_. It is conceivable that, despite the ROS-dependent inhibition, SERCA2B is able to cope with such decreased Ca^2+^ signal, thereby erasing the differences existing between LZDF and OZDF animals. It remains to be assessed whether specific blockers of IK_Ca_ and/or SK_Ca_ channels exert a similar impact of the Ca^2+^ response to 300 µM ATP. In addition, it will have to be evaluated whether resting membrane potential, which is mainly controlled by inward rectifier K^+^ channels in rat aortic endothelial cells, is altered by type 2 diabetes mellitus.

### 3.4. How the Impairment of SERCA2B Activity Could Result in Endothelial Dysfunction in Type 2 Diabetes Mellitus

Earlier studies have demonstrated that the Ca^2+^ handling machinery is severely impaired in the cardiovascular system of OZDF rats, but these studies were conducted on vascular smooth muscle cells [73,74,75] and cardiomyocytes [62,76], rather than on vascular endothelial cells [70]. In this latter study, the Ca^2+^ response to acetylcholine was greatly enhanced in coronary artery endothelial cells of OZDF rats due to the up-regulation of small- and intermediate-conductance Ca^2+^-activated K^+^ channels [70]. However, the increased amplitude of the plateau phase could reflect the impairment of SERCA activity, which does not fully sequester Ca^2+^ into ER lumen, thereby boosting the increase in [Ca^2+^]_i_. These findings further support the notion that remodeling of the endothelial Ca^2+^ toolkit described in in vitro endothelial cells cultured under high glucose conditions differs from that reported in situ. For instance, SOCE was up-regulated in bovine aortic endothelial cells treated with high glucose for 72 h [51], while it was down-regulated in native aortic endothelium (present study). The increase in the Ca^2+^ response to supramaximal concentrations of physiological agonists, such as ATP, could be involved in the impairment of endothelial signaling associated with type 2 diabetes mellitus [50]. For instance, exaggerated Ca^2+^ signals could further enhance NOX activation, thereby resulting in a burst of anion superoxide (O2•−) production and worsening endothelial dysfunction [24,77]. Furthermore, an aberrant increase in [Ca^2+^]_i_ causes the up-regulation of the Ca^2+^-dependent xanthine oxidase and induces ROS overproduction in human umbilical vein endothelial cells [78]. Finally, a recent study revealed that cytosolic Ca^2+^ overload leads to Ca^2+^ accumulation within the mitochondrial matrix, thereby inducing O2•− production and endothelial dysfunction [79]. It should, however, be pointed out that, in addition to type 2 diabetes mellitus, OZDF rats develop additional cardiovascular risk factors, including obesity and hyperlipidemia, that also have the potential to affect the Ca^2+^ signaling toolkit [80,81]. Future work will have to assess whether type 2 diabetes mellitus still impairs the endothelial Ca^2+^ machinery in the absence of these comorbidities.

## 4. Materials and Methods

### 4.1. Animals

All the experimental procedures on animals were performed according to protocols approved by the Animal Care and Use Committee of the Benemerita Universidad Autonoma de Puebla, identification code: BERRSAL71, 18-05-2017. Every effort was made to minimize the number of animals used and to ensure minimal pain and/or discomfort. Experiments were carried out in male Zucker Diabetic Fatty (ZDF) rats (2–3 months old) from Charles River Laboratories, California, USA Throughout the text, diabetic-obese ZDF rats (ZDF-Leprfa/fa) will be designated as OZDF rats, and lean controls, non-obese non-diabetic ZDF (ZDF-Lepr+/+) as LZDF. The rats were kept at the University Animal Core Facilities under controlled environmental temperature and, exposed to light-dark cycles of 12 h, with ad libitum consumption of water and Purina 5008 chow.

### 4.2. Morphometric Parameters

On the day of the experiment, we proceeded to measure the body mass (weight), length (distance from the tip of the nose to the base of the tail) and the abdominal circumference using a measuring tape. The BMI was calculated using the following equation:

(1) $$\mathrm{BMI}=\;\frac{\mathrm{Body}\;\mathrm{mass}\;(g)}{{\mathrm{Length}}^{2}\;{\left(\mathrm{cm}\right)}^{2}}$$

### 4.3. Oral Glucose Tolerance Test and Insulin Response, Insulin Tolerance Test and Insulin Resistance

The oral glucose tolerance (OGTT) test was performed on rats that had been fasted 4 h, before a glucose anhydrous solution (2 g/kg BW) was administered orally, glucose was measured from the vein of the tail. Then, the glucose loading was administered, and glucose plasmatic concentration was determined at 30, 60, 90 and 120 min from the vein of the tail. The glucose concentration was measured by a commercial kit (Spinreact, Spain) and an automatized analyzer A-15 (BioSystem, Jalisco, Mexico). At the same time (0, 30, 60, 90 and 120 min), plasmatic insulin was quantified by a commercial kit (Diagnostica International Company, Jalisco, Mexico), with the resulting antibody-antigen complex assessed at 415 nm in a Stat Fax 2600 plate reader (WinerLab Group, Rosário, Argentina). Insulin concentrations were obtained from a standard curve ranging from 0 to 200 µUI/mL.

Five days after the oral glucose tolerance test. On rats that had been fasted 4 h, an insulin tolerance test (ITT) was performed. The animals were intraperitoneally challenged with a dose of 0.75  U/kg BW of human insulin (Humulin 70/30; Lilly, Indianapolis, IN, USA). This insulin is a combination of human insulin isophane suspension and human insulin (rDNA), which combines intermediate-acting insulin with the more rapid onset of action of regular human insulin. The pharmacologic effect begins at approximately 50 min (range: 30 to 90 min); thus, it is ideal insulin for realizing an ITT. Blood samples were drawn from the tail vein at different time points (0, 15, 30, 60 and 90 min), and glucose levels were determined as described previously.

Finally, the insulin resistance was evaluated using the homeostasis model assessment insulin resistance (HOMA-IR). The mathematical model was realized according to the report by [82].

### 4.4. Dissection of the Aorta, Blood and Epididymal Fat Samples

ZDF rats, fasting for 6 h, were anesthetized with intraperitoneal ketamine-xylazine solution, 0.2 mL per 100 g of weight; subsequently they underwent to an anterior thoracotomy to expose the aortic arch and the heart. A blood sample (5 mL) was obtained with 5 mL syringes through the inferior vena cava to carry out the biochemical tests. Blood samples were transferred to vacutainer tubes without anticoagulant, and clot formation was allowed for 10 min. Once the blood was coagulated, it was centrifuged at 10,000 revolutions per minute (r/min) for 15 min at controlled room temperature (22–23 °C). The serum was then extracted from the globular package with a micropipette and placed in 1 mL Eppendorf tubes, which were stored at 4 °C to be taken to a clinical analysis laboratory, where total cholesterol, triglycerides, HDL-C, LDL-C and VLDL concentrations were quantified.

The thoracic and abdominal aorta were dissected out and perfused with physiological salt solution (PSS). The vessel was placed in a Petri dish with PSS. Using a stereomicroscope (Nikon SMZ-2T, Tokyo, Japan), the connective and fatty tissues surrounding the aorta were removed. Subsequently the aorta was cut into ~5 mm wide rings, stored in physiological salt solution (PSS) at controlled room temperature (22–23 °C), and used within 5 h.

Finally, all the epididymal fat surrounding both testes was accurately removed and weighed.

### 4.5. Solutions

PSS had the following composition (in mM): 150 NaCl, 6 KCl, 1.5 CaCl_2_, 1 MgCl_2_, 10 Glucose, 10 HEPES. In Ca^2+^-free solution (0Ca^2+^), Ca^2+^ was substituted with 2 mM NaCl, and 0.5 mM ethylene glycol-bis(2-aminoethylether)-N,N,N′,N′-tetraacetic acid (EGTA) was added. Solutions were titrated to pH 7.4 with NaOH. Aortic rings were bathed in 0Ca^2+^ for no longer than 90 s. Control experiments have demonstrated that such a short pre-incubation period is not able deplete intracellular Ca^2+^ stores (Berra-Romani et al., 2008). For high K^+^-containing solution (KCl 90 mmol/L), osmolality was corrected by equimolar reduction of NaCl.

### 4.6. Intracellular Ca^2+^ Concentration Measurements

This technique for evaluating changes in [Ca^2+^]_i_ in intact endothelium has been previously described [21,36,39,83]. Briefly, using a microdissection scissors, the aortic rings were carefully cut to open them and obtain aortic strips with intact endothelium. The aortic strips were loaded with 16 µmol Fura-2/AM for 60 min at room temperature, washed and fixed (with the luminal face up) to the bottom of a Petri dish covered by inert silicone (Silgard ^®^ 184 Silicone Elastomer, Down Corning, MI, USA) by using four 0.4 mm diameter pins. In situ endothelial cells were visualized by an upright epifluorescence Axiolab microscope (Carl Zeiss, Oberkochen, Germany), equipped with a Zeiss × 40 Achroplan objective (water-immersion, 2.05 mm working distance, 1.0 numerical aperture). Endothelial cells were excited alternately at 340 and 380 nm, and the emitted light was detected at 510 nm. The exciting filters were mounted on a filter wheel (Lambda 10, Sutter Instrument, Novato, CA, USA). Custom software, working in the LINUX environment, was used to drive the camera (Extended-ISIS Camera, Photonic Science, Millham, UK) and the filter wheel, and to measure and plot on-line the fluorescence from 52–126 “regions of interest” enclosing one single cell. [Ca^2+^]_i_ was monitored by measuring, for each regions of interest, the ratio of the mean fluorescence emitted at 510 nm when exciting alternatively at 340 and 380 nm (termed Ratio (F340/F380)). An increase in [Ca^2+^]_i_ causes an increase in the Ratio (F340/F380). Ratio (F340/F380) measurements were performed and plotted on-line every 3 s. Ratio (F340/F380) values are expressed as arbitrary units (A.U.). The experiments were performed at controlled room temperature (22–23 °C) to limit time-dependent decreases in the intensity of the fluorescence signal.

### 4.7. Data Analysis

For each protocol, data were collected from at least three rats under each condition. The amplitude of the peak Ca^2+^ response to ATP was measured as the difference between the ratio at the peak and the mean ratio of 1 min baseline before the peak. The amplitude of the plateau phase was measured as the difference between the ratio at 600 s after the application of ATP 20 µM and ATP 300 µM, respectively, and the mean ratio of 1 min baseline before the peak. The duration of the Ca^2+^ response to ATP was measured as the time it takes the Ca^2+^ signal to be reduced at 90% (early), 60% (intermediate) and 30% (late) of the initial Ca^2+^ peak amplitude. The area under the curve (AUC) was measured by calculating the integral of each Ca^2+^ tracing from when the ATP is applied until it is removed. Data are expressed as mean ± standard error (SE). Non-Gaussian data (identified using the D’Agostino and Pearson omnibus normality test (*p* < 0.05)), were statistically analyzed using nonparametric Mann-Whitney test. For normal data an unpaired Student’s *t*-test was used. A *p* value < 0.05 was considered statistically significant.

### 4.8. Chemicals

Fura-2/AM was obtained from Molecular Probes (Molecular Probes Europe BV, Leiden, The Netherlands). SEA0400 was obtained from Tocris Bioscience (Bristol, UK). All other chemicals were purchased from Sigma (Sigma-Aldrich Quimica, Toluca, Estado de México, México).

### 4.9. Immunohistochemistry

To detect SERCA expression, aortic tissues were extracted from OZDF or LZDF rats and were fixed in 4% paraformaldehyde solution, washed and incubated with PBS 0.25% Triton X-100 and blocked with PBS 3% bovine serum albumin followed by incubation at 4 °C overnight with 2 mg/mL Anti-SERCA2 ATPase antibody (ab2861, Abcam); washed and incubated with 2 mg/mL Alexa Fluor488-conjugated anti-mouse IgG antibody (6787, Abcam) for 1 h at room temperature. Additionally, the cell nuclei were counter-stained with 0.001% Hoescht 33342 solution. The tissues were mounted in slides and fluorescence was detected in 2 μm sections in a Zeiss Observed Z1 inverted microscope equipped with an Axiocam MRm camera and an Apotome illumination system with a 63X oil immersion objective (Carl Zeiss Microscopy, New York, United States).

## 5. Conclusions

In conclusion, this report demonstrated for the first time that the Ca^2+^ handling machinery is altered in native endothelium of excised aorta from OZDF rats. The Ca^2+^ response to physiological stimuli is remarkably enhanced when agonist concentration is elevated within the high micromolar range. Rewiring of the endothelial Ca^2+^ toolkit involves the ROS-dependent down-regulation of SERCA activity, which results in the increase of the amplitude of plateau phase, thereby prolonging the duration of the Ca^2+^ signal. This remodeling of the endothelial Ca^2+^ machinery could contribute to explaining how type 2 diabetes leads to endothelial dysfunction.

## Figures and Tables

**Table 1:** Somatic and biochemical parameters of ZDF rats. The values represent the mean ± SE (standard error). Data were compared using Student’s *t*-test with a minimum significance value of 0.05 (*p* value). The * represent the significant differences observed when compare the OZDF vs LZDF group. Analysis of somatic parameters was performed with a n of 11 rats for the LZDF group and 14 rats for the OZDF group. For the biochemical analysis, 5 rats of each group were used. BMI (body mass index), HDL-C (high-density lipoprotein cholesterol), LDL-C (low-density lipoprotein cholesterol), VLDL (very low-density lipoprotein).

**Somatic Parameters**	**LZDF (*n* = 11)**	**OZDF (*n* = 14)**
Weight (g)	309.6 ± 6.03	529 ± 8.16 *
Length (cm)	22.41 ± 0.29	23.5 ± 0.33
Abdominal circumference (cm)	13.21 ± 0.29	17.85 ± 0.36 *
BMI	0.59 ± 0.009	0.93 ± 0.016 *
Epididymal fat (g)	3.32 ± 0.12	15.71 ± 0.62 *
**Biochemical Parameters**	**LZDF (*n* = 5)**	**OZDF (*n* = 5)**
Total Cholesterol (mg/dL)	90.83 ± 12.22	133 ± 11.82 *
HDL-C (mg/dL)	61.6 ± 3.02	72.06 ± 8.22
LDL-C (mg/dL)	26.48 ± 12.09	35.64 ± 13.26
VLDL (mg/dL)	11.53 ± 3.62	34.53 ± 3.95 *
Triglycerides (mg/dL)	42 ± 10.35	186.1 ± 23.04 *

## Abbreviations

A.U.	Arbitrary units
AUC	Area under de curve
ATP	Adenosine triphosphate
BMI	Body mass index
HOMA-IR	Homeostasis model assessment insulin resistance
ITT	Insulin tolerance test
[Ca^2+^]_i_	Intracellular Ca^2+^ concentration
CE	Carboxyeosin
CPA	Cyclopiazonic acid
CV	Cardiovascular
ER	Endoplasmic reticulum
HDL-C	High-density lipoprotein cholesterol
InsP_3_	Inositol-1,4,5-trisphosphate
KBR	KBR-79433
LDL-C	Low-density lipoprotein cholesterol
LZDF	Lean Zucker Diabetic Fatty rats
NCX	Na+/Ca^2+^ exchanger
OGTT	Oral glucose tolerance test
OZDF	Obese Zucker Diabetic Fatty rats
PMCA	Plasma membrane Ca^2+^-ATPase
PSS	Physiological salt solution
SEA	SEA0400
SERCA	Sarco-endoplasmic reticulum Ca^2+^-ATPase
SOCE	Store-operated Ca^2+^ entry
TG	Thapsigargin
VLDL	Very low-density lipoprotein
